# Role of inflammation in alcohol-related brain abnormalities: a translational study

**DOI:** 10.1093/braincomms/fcab154

**Published:** 2021-07-16

**Authors:** Anastasia Lanquetin, Sophie Leclercq, Philippe de Timary, Shailendra Segobin, Mikaël Naveau, Laurent Coulbault, Paola Maccioni, Irene Lorrai, Giancarlo Colombo, Denis Vivien, Marina Rubio, Anne-Lise Pitel

**Affiliations:** 1 Normandie Univ, UNICAEN, INSERM, PhIND “Physiopathology and Imaging of Neurological Disorders”, Institut Blood and Brain @ Caen-Normandie, Cyceron, 14000 Caen, France; 2 Institute of Neuroscience and Louvain Drug Research Institute, UCLouvain, Université Catholique de Louvain, Brussels, Belgium; 3 Normandie Univ, UNICAEN, PSL Université Paris, EPHE, INSERM, U1077, CHU de Caen, GIP Cyceron, Neuropsychologie et Imagerie de la Mémoire Humaine, 14000 Caen, France; 4 Normandie Univ UNICAEN, CNRS, UMS 3408, GIP Cyceron, Caen, France; 5 Caen University Hospital, Biochemistry Department, Normandie University, UNICAEN, EA 4650, Caen, France; 6 Neuroscience Institute, Section of Cagliari, National Research Council of Italy, 09042 Monserrato, CA, Italy; 7 Department of Clinical Research, CHU Côte de Nacre, Caen 14000, France; 8 Institut Universitaire de France (IUF), Paris 75231, France

**Keywords:** alcohol use disorders, inflammation, brain, MRI

## Abstract

Brain abnormalities observed in alcohol use disorder are highly heterogeneous in nature and severity, possibly because chronic alcohol consumption also affects peripheral organs leading to comorbidities that can result in exacerbated brain alterations. Despite numerous studies focussing on the effects of alcohol on the brain or liver, few studies have simultaneously examined liver function and brain damage in alcohol use disorder, and even fewer investigated the relationship between them except in hepatic encephalopathy. And yet, liver dysfunction may be a risk factor for the development of alcohol-related neuropsychological deficits and brain damage well before the development of liver cirrhosis, and potentially through inflammatory responses. The use of animal models enables a better understanding of the pathophysiological mechanisms underlying liver–brain relationships in alcohol use disorder, and more particularly of the inflammatory response at the tissue, cerebral and hepatic levels. The objective of this translational study was to investigate, both in alcohol use disorder patients and in a validated animal model of alcohol use disorder, the links between peripheral inflammation, liver damage and brain alterations. To do this, we conducted an *in vivo* neuroimaging examination and biological measures to evaluate brain volumes, liver fibrosis and peripheral cytokines in alcohol use disorder patients. In selectively bred Sardinian alcohol-preferring rats, we carried out *ex vivo* neuroimaging examination and immunohistochemistry to evaluate brain and liver inflammatory responses after chronic (50 consecutive weeks) alcohol drinking. In recently abstinent and non-cirrhotic alcohol use disorder patients, the score of liver fibrosis positively correlated with subcortical regions volumes (especially in right and left putamen) and level of circulating proinflammatory cytokines. In Sardinian alcohol-preferring rats, we found macrostructural brain damage and microstructural white matter abnormalities similar to those found in alcohol use disorder patients. In addition, in agreement with the results of peripheral inflammation observed in the patients, we revealed, in Sardinian alcohol-preferring rats, inflammatory responses in the brain and liver were caused by chronic alcohol consumption. Since the liver is the main source of cytokines in the human body, these results suggest a relationship between liver dysfunction and brain damage in alcohol use disorder patients, even in the absence of major liver disease. These findings encourage considering new therapeutic strategies aiming at treating peripheral organs to limit alcohol-related brain damage.

## Teaser

Abbreviated summary

Lanquetin et al. report that systemic inflammation correlates with liver and brain abnormalities in alcohol use disorder patients. In alcohol-exposed rats, brain damage is associated with neuroinflammatory responses and liver inflammation. Chronic alcohol consumption results in peripheral inflammation, which alters the inflammatory status of the brain and liver in cascade.

## Graphical Abstract

**Graphical Abstract:**
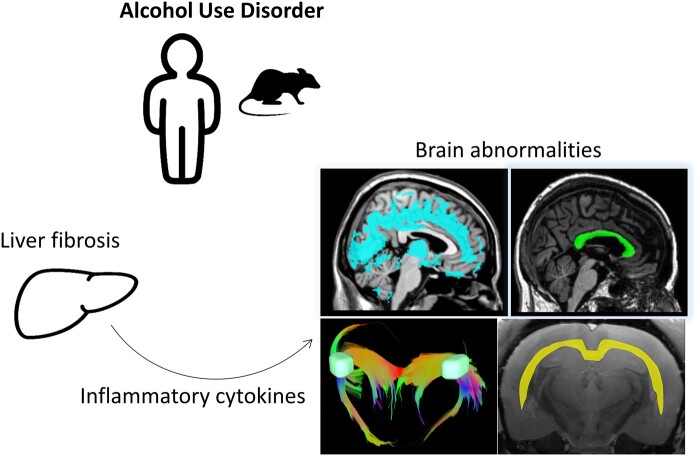

## Introduction

Alcohol use disorder (AUD) is a chronic relapsing brain disease, which represents a major public health problem affecting millions of people around the world.^1^ The most recent version of the Diagnostic and Statistical Manual of Mental Disorders (DSM-5^2^) longer considers AUD as a categorical disease (abuse versus dependence, as in previous version, DSM-IV),^3^ but offers a dimensional approach with a spectrum of severity ranging from mild, moderate, to severe AUD. DSM-5 also acknowledges alcohol-related neurocognitive disorders in agreement with the literature that frequently reports altered brain structure and neuropsychological impairments in AUD patients.^4–6^

Chronic alcohol consumption is indeed associated with brain alterations that can be revealed *in vivo* by MRI. In AUD patients, grey matter (GM) structural abnormalities can affect cortical and subcortical regions. The frontal cortex seems to be especially sensitive to chronic and heavy alcohol consumption,^7^ but shrinkage has also been observed in temporal, parieto-occipital cortex and in the cerebellum.^8^ In cortical and subcortical regions, atrophy of the hippocampus,^9^ mamillary bodies, cingulate cortex and thalami^8^^,^^10^ have also been reported.

Regarding the white matter (WM), MRI studies revealed a thinning of the corpus callosum.^11^^,^^12^ Diffusion tensor imaging (DTI) and in particular the measure of fractional anisotropy (FA), are specific MRI techniques that explore non-invasively WM microstructure and integrity (myelination or axonal integrity^13^^,^^14^). DTI studies conducted in AUD patients have shown microstructural alterations in the fornix and corpus callosum,^6^^,^^15^^,^^16^ even when no macrostructural MRI abnormalities were observed.^17^^,^^18^ While structural brain abnormalities are currently relatively well characterized in AUD patients, the pathophysiology underlying these brain damages remain unclear and need being explored, in particular by using animal models.

Preclinical neuroimaging studies suggested that chronic alcohol drinking resulted in ventricular expansion, hippocampus shrinkage,^19^^,^^20^ and decrease in volume and FA in fibre tracts of the corpus callosum and fornix^15^ in rats.

Our groups are interested in testing the possibility that damages developed at the level of peripheral organs participate to the development of the brain.^21^^,^^22^ Hepatic encephalopathy (HE) induced by chronic alcohol consumption is the extreme example of brain and liver interaction. However, long before the development of a full-blown HE, altered liver function may affect brain structure and function. Ritz et al.^22^ for instance observed that liver fibrosis is related and may partially explain executive dysfunction in AUD patients without clinically detectable HE. Junghanns et al.^23^ also observed that gamma-glutamyl transferase (GGT) levels were related to mental flexibility abilities. These results indicate that liver dysfunction may predict the severity of executive impairments in AUD patients, suggesting the existence of a liver brain communication. Peripheral inflammation could be one of these pathways of communication, where the release of cytokines could contribute to neuroinflammation.^21^^,^^24^

Preclinical and human *post-mortem* studies have suggested neuroinflammation as an important pathogenic process participating to alcohol-induced brain damage,^25^ with a pivotal role of microglial activation.^26–28^ However, the study of neuroinflammation in AUD patients by PET using translocator protein-18 kDa (TSPO) ligand (used to evaluate microglial activation *in vivo*) has shown controversial results, with either lower or equal levels of neuroinflammation in AUD patients compared to controls.^29–31^ Very recently, De Carvalho et al.^32^ showed, for the first time, an increased transcription of *TSPO*, *HDAC2* and *HDAC6* in the amygdala of AUD patients suggesting a particular vulnerability of this region to neuroinflammation. Circulating cytokines can be used to evaluate the inflammatory state in AUD patients. Cytokines are considered important mediators of the systemic-brain communication, as they can reach the CNS and induce neuroinflammation that is associated with changes in mood, cognition and drinking behaviour.^33^

The objectives of this translational study were to investigate, both in AUD patients and in selectively bred alcohol-preferring rats, the links between peripheral inflammation, liver damage and brain alterations. To do so, we have examined grey and white brain matter abnormalities, liver damage, and peripheral cytokines levels in a cohort of recently detoxified AUD patients as well as in chronically alcohol drinking Sardinian alcohol-preferring (sP) rats.

Our results show that, in both the human and the animal model, alcohol consumption provokes liver and brain inflammatory responses that are correlated with brain abnormalities, in the absence of advanced liver damages.

## Material and methods

### Clinical data

#### Participants

Forty-one participants were included in this study: 25 patients with AUD and 16 healthy controls (HCs). None of them had a history of neurological pathology, endocrinal nor infectious diseases, depression [assessed using the Beck Depression Inventory (BDI)]^34^; nor other forms of substance use disorder (except tobacco). All participants were informed about the study approved by the local ethics committee of Caen University Hospital (CPP Nord Ouest III, no. IDRCB: 2011‐A00495‐36) prior to their inclusion and provided their written informed consent. The study was carried out in line with the declaration of Helsinki (1964).

Clinicians recruited AUD patients while they were receiving withdrawal treatment as inpatients at Caen University Hospital. AUD patients met ‘alcohol dependence’ criteria according to the DSM‐IV‐TR^3^ and ‘severe AUD’ criteria according to the DSM‐5^2^ for at least 5 years. At inclusion and evaluation, none of them presented physical symptoms of alcohol withdrawal as assessed by Cushman's scale^35^ (score ≤ 2) nor were under medication by benzodiazepines. Alcohol history of the AUD patients is described in Table 1.

**Table 1:** Demographical and clinical description of the healthy controls (HCs) and alcohol use disorder patients (AUD)

		AUD *N* = 25	HC *N* = 16	*P*-value
Demography	Age (years)	46.84 (8.586)	46.38 (6.531)	0.8543
	Men/Women Ratio	20/5	13/3	0.8718^*^
	Education (years of schooling)	12.04 (1.881)	11.69 (2.33)	0.5816
Clinical variables	BMI	23.28 (0.586)	24.88 (0.8945)	0.1278^a^
	DRS (total score/144)	135.4 (8.675)	141.4 (2.159)	**0.0021**
	BDI	10.68 (7.454)	3.625 (3.008)	**0.0006**
	STAI-A	29.74 (10.69)^b^	27.50 (7.220)	0.6555
	STAI-B	43.39 (12.07)^b^	33.88 (6.365)	**0.0174**
	AUDIT	29.08 (6.788)	2.875 (1.668)	**<0.0001**
	Alcohol misuse (number of years)	19.667 (8.91)	NA	**/**
	Daily alcohol consumption (units)	19.864 (8.91)	NA	**/**
	Length of sobriety (days)	11.42 (4.94)	NA	**/**

AUDIT, Alcohol Use Disorders Identification Test; ASAT/ALAT, transaminases; BDI, Beck Depression Inventory; DRS, Mattis Dementia Rating Scale; GGT, Gamma-glutamyl transferase; STAI, State-Trait Anxiety.

a *P*-value for *N* = 15 HC, one HC subject being an outlier (BMI = 35).

b Two missing data.

* Chi^2^.

HCs were recruited locally and to match the demographics of the AUD patients. They were interviewed with the Alcohol Use Disorders Identification Test^36^ (AUDIT) to ensure that they did not meet the criteria for alcohol abuse (AUDIT < 7 for men and <6 for women). None of the controls had a BDI score > 29 and a score <136 on the Dementia Rating Scale^37^ (DRS-2).

AUD patients and HC were age‐, sex‐, and education‐matched (Table 1).

#### Biological assessment

Fasting blood samples were collected for all participants, either at inclusion (HC) or the day after admission to hospital (AUD) (24 h) to ensure that patients were free from alcohol and to minimize the potential confusing effect of withdrawal.

Liver function was assessed using the level of GGT and the aspartate transaminase (ASAT)/alanine transaminase (ALAT) ratio (a ratio > 1 suggested alcoholic liver disease^38^). We used the FibroMeter^®^ for the non-invasive diagnosis of alcoholic liver fibrosis based on the measure of 4 blood markers: alpha 2-macroglobulin, platelets, prothrombin ratio and hyaluronic acid.^39^ This yielded a fibrosis score (ranging from 0 to 1) and a percentage of fibrosis area (ranging from 0 to 100%).

Serum levels of the four inflammatory cytokines interleukin 8 (IL-8), tumour necrosis factor (TNF), monocyte chemoattractant protein-1 and macrophage inflammatory protein 1β were assayed by a multiplex cytokine assay (Human Bio-Plex; Bio-Rad Laboratories Inc., Hercules, CA, USA) according to the instructions from the manufacturer.

#### Magnetic resonance imaging data acquisition

Brain imaging examinations were conducted in 16 HCs and 25 AUD patients.

A high‐resolution T_1_‐weighted anatomical image was acquired for each subject on a Philips Achieva 3 T scanner (Philips Healthcare/Philips Medical Systems International B.V., Eindhoven, the Netherlands) using a three‐dimensional fast‐field echo sequence (sagittal; repetition time, 20 ms; echo time, 4.6 ms; flip angle, 10°; 180 slices; slice thickness: 1 mm; field of view, 256 × 256 mm^2^; matrix, 256 × 256).

Regarding DTI, 70 slices (thickness: 2 mm, no gap) were acquired axially using a diffusion‐weighted imaging spin echo sequence [32 directions at b = 1000 s/mm^2^, repetition time = 10 000 ms, echo time = 82 ms, flip angle = 90°, field of view = 224 × 224 mm^2^, matrix = 112 × 112, and in‐plane resolution = 2 × 2 mm^2^; 1 no–diffusion‐weighted image (DWI) at b = 0 s/mm^2^ was also acquired].

#### Magnetic resonance imaging data processing

The volumetric MRI data of GM and WM were analysed using the Statistical Parametric Mapping software (SPM12; Wellcome Department of Cognitive Neurology, Institute of Neurology, London, UK). Pre-processing steps included segmentation of the MRI data into GM, WM and CSF, and spatial normalization to the Montreal Neurological Institute (MNI) template (voxel size = 1.5 mm^3^, matrix = 121 × 145 × 121). The resulting normalized images were modulated by the Jacobian determinants to preserve brain volume, and smoothed by a Gaussian kernel of 8 mm full width at half maximum (FWHM). GM (and WM) measures reflect cerebral macrostructure and numerically corresponds to the mean GM (or WM) signal per unit volume for each significant cluster.

The DTI data were processed as previously described by Segobin et al.^40^ to create FA maps. Numerically, FA values vary between 0 and 1. Generally, the higher the FA value, the better the microstructural integrity of the fibre within that voxel. FA is assumed to be a structural biomarker that depicts WM disruption involving myelin, cytoskeleton and the axons’ microtubule system.^41^

A GM mask was obtained taking the unmodulated GM images of HC normalized to the MNI space, averaging them, and thresholding the resultant mean image at 0.5. The WM mask was obtained via the same procedure as for the GM mask, that is, by using unmodulated WM images from the HC group in MNI space, averaging them and thresholding the resultant mean image at 0.5. The same WM mask was used when analysing both, WM volumes and FA values respectively, while GM mask was used when analysing GM volumes.

#### Statistical analysis

##### Biological assessment of the AUD patients

The normality of the distribution of the laboratory measures for the AUD and HC groups was examined using the Shapiro–Wilk test. Mann–Whitney tests were then used to compare the two groups for measures of liver function and inflammatory cytokines.

##### Pattern of brain alterations and correlations with biological variables

Voxel‐based *t*-tests were conducted in SPM12 to compare HC and AUD on GM volume, WM volume and WM integrity (FA values). The *t*-tests were performed individually for each imaging measurement. Results are reported at *P* < 0.05 (FWE-corrected) and at *P* < 0.001 (uncorrected for multiple comparisons) with a minimal cluster size (*k*) of 60 voxels (200 mm^3^ for volumetric analysis; 60 mm^3^ for DTI analysis).

Voxel-based multiple linear regressions were then conducted in SPM12 to examine, only in AUD patients, the relationships between GM volume, WM volume and WM integrity (FA values) on the one hand, and fibrosis (score and area) and elevated levels of cytokines on the other hand. Results are reported at *P* < 0.001 (uncorrected for multiple comparisons) with a minimal cluster size (*k*) of 60 voxels (200 mm^3^). The regressions were conducted individually for each imaging measurement.

Significant clusters of GM were labelled using the Harvard–Oxford cortical and subcortical structural atlases implemented in FSL (https://fsl.fmrib.ox.ac.uk/fsl/fslwiki/Atlases). WM regions and tracts were manually labelled using the MRI Atlas of Human White Matter.^42^

### Preclinical data

#### Animal subjects

The *in vivo* portion of the study with sP rats was performed at the Neuroscience Institute, Section of Cagliari, National Research Council of Italy. The experimental procedures employed in this study fully complied with European Directive no. 2010/63/EU and subsequent Italian Legislative Decree no. 26, 4 March 2014, on the ‘Protection of animals used for scientific purposes’.

We used male, 95th‐generation sP rats that were approximately 75 days old at the start of the study. Rats were individually housed in standard plastic cages with wood chip bedding. Single‐cage housing started 15 days before exposure to alcohol. The animal facility was under an inverted 12:12‐h light/dark cycle (lights on at 9:30 pm) at a constant temperature of 22 ± 2°C and a relative humidity of approximately 60%. Regular food pellets (Mucedola, Settimo Milanese, Italy) were always available. Alcohol was offered under the standard, home‐cage 2‐bottle choice regimen between an alcohol solution (10% in tap water, v/v) and tap water with unlimited access for 24 h/d. Under this procedure, sP rats usually consume daily approximately 6 g/kg pure alcohol with relatively steady intakes over time, modelling excessive alcohol drinking in humans.^43^ The left–right position of the two bottles was randomly interchanged to avoid the development of position preference. Alcohol and water intake were recorded once weekly. Alcohol-drinking rats (*n* = 6) were exposed to the ‘alcohol *vs* water’ choice regimen for 50 consecutive weeks. Conversely, control rats (*n* = 6) were exposed to 2 bottles containing tap water.

At the end of the 50-week period of alcohol exposure, rats were killed by guillotine. Alcohol and water bottles were removed immediately before killing. Heads (after removal of the skin and muscles) and liver samples were immediately fixed in a solution of 2% paraformaldehyde and 0.2% picric acid phosphate buffer for 48 h. Samples were then individually placed in 50 ml falcon and rinsed with a solution of PBS with 20% sucrose followed by PBS with azide (0.01%) and stored at 4°C for further analysis.

#### *Ex vivo* brain MRI acquisitions

Imaging was carried out on a Pharmascan 7 T/12 cm system using surface coils (Bruker, Germany). Anatomical images were acquired with high resolution sequence T2 RARE (Rapid acquisition with repetition enhancement) in 3D with the following parameters: TE 40 ms, TR 4500 ms, resolution 0.1 × 0.1 × 0.1 mm^3^, thickness 0.1 mm, field of view 3.2 × 3.2 × 2.56, acquisition time 3h50. DTI data were acquired with the following parameters: TR 3500 ms, TE 23 ms, 32 diffusion directions, b = 1048 s/mm^2^ 64 horizontal slices were planned for every subject (field of view = 32 × 32 mm^2^, in-plane resolution = 0.2 × 0.2 mm^2^, slice thickness = 0.4 mm).

#### MRI processing

Volumetric analyses were performed using ImageJ (http://imagej.nih.gov/ij), with manual delimitation of regions of interest (ROI) from one blinded experimenter (AL) and automatic calculation of these regional areas. To correctly delimit ROIs, we used the Paxinos–Watson rat brain atlas.^44^

DTI analyses were conducted with the software DSI Studio (http://dsi-studio.labsolver.org). For each subject, the following parameter maps were computed: FA, mean diffusivity (MD), axial diffusivity (AD) and radial diffusivity (RD). Whole brain tractography was calculated using a deterministic, tensor-based approach with the following parameters: FA > 0.20 and angle threshold 45°.

#### Immunohistochemistry

Brain and liver portions were frozen in Tissue-Tek (Miles Scientifix, Naperville, IL, USA). Cryostat-cut sections (10 µm) were collected on poly-lysine slides and stored at −80°C before processing.

Sections were co-incubated overnight with goat anti-rat Iba1 (1:1000, abcam ab5076), chicken anti-rat glial fibrillary acidic protein (GFAP) (1:2000, abcam ab4674), goat anti-rat collagen-IV (1:1000, Southern biotech 1340), rabbit anti-rat cleaved caspase 3 (1:1000, Cell signalling 9664-5), rabbit anti-rat myeloperoxidase (1:200, abcam ab9535). Primary antibodies were revealed using Fab’2 fragments of donkey anti-goat linked to CY3 or FITC, anti-rabbit linked to CY3 or FITC, anti-chicken linked to CY5 (1:600 Jackson ImmunoResearch, West Grove, USA). Washed sections were coverslipped with antifade medium containing DAPI. Epifluorescence images were digitally captured using a LEICA DM6000 epifluorescence microscope-coupled coolsnap camera, visualized with Leica MM AF 2.2.0 software (Molecular devices, USA) and further processed using ImageJ. Specificity controls were performed by not adding primary antibodies.

Fluorojade C staining protocol was performed as previously described.^45^

Liver sections were taken from the left lateral lobe in all animals. Coronal brain sections (10 µm-thick) containing the corpus callosum, cortex and/or hippocampus (coordinates: bregma −2 to −4.8 mm according to Paxinos–Watson^44^) were collected on gelatinized slices. For all the structures of interest, at least 5 images in 1 or 2 different sections per animal were analysed.

#### Statistical analysis

Preclinical data were analysed using Mann–Whitney’s tests to compare alcohol-drinking versus control (i.e. water-exposed) sP rats. *P*-values <0.05 were considered statistically significant.

### Data availability

All data and materials used within this study will be made available, upon reasonable request, to research groups wishing to reproduce/confirm our results.

## Results

### Clinical data

#### Pattern of brain alterations in AUD patients

##### GM volume

**Figure 1:**
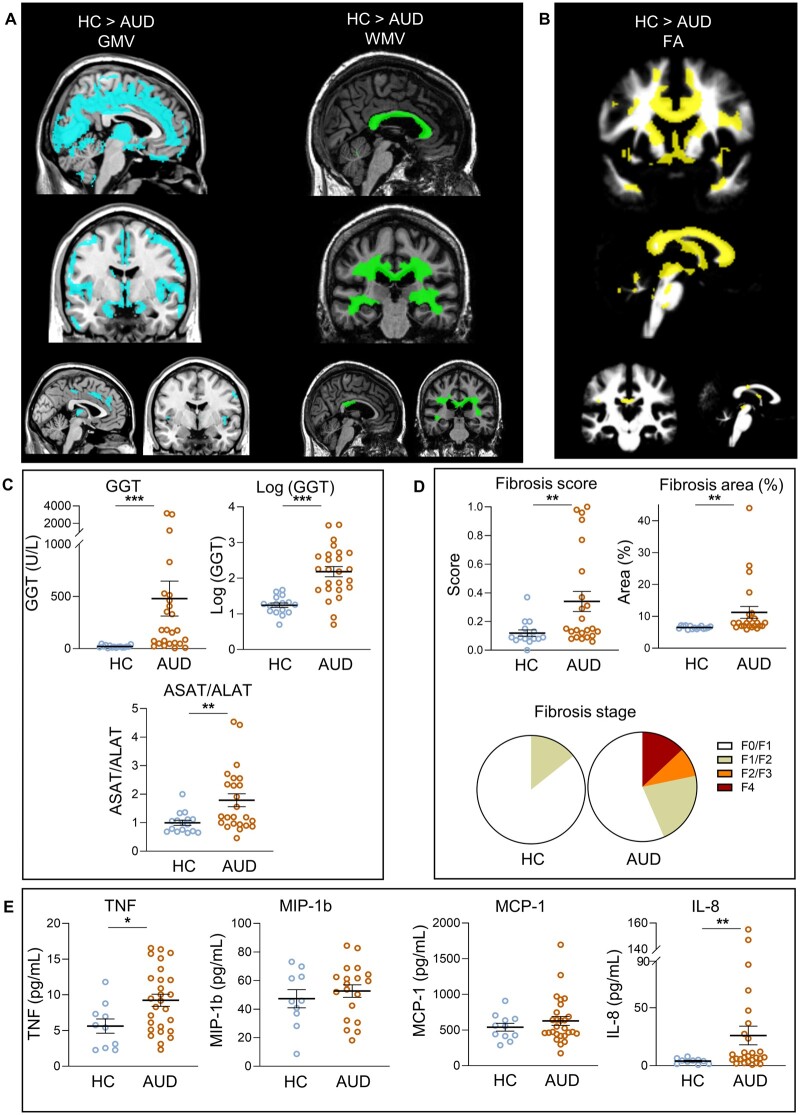
**Brain alterations, hepatic dysfunction, liver fibrosis and peripheral inflammation in HC and in AUD patients.** (**A**) AUD patients have significant grey matter (GM), and white matter (WM), shrinkage, as well as altered WM integrity (**B**) compared to healthy controls (HCs). Larger images indicate a *P*‐value of <0.001 uncorrected, and smaller images display the results using a restrictive *P* < 0.05 corrected for FWE to highlight the most significant regions (**C**) AUD patients had hepatic dysfunction with elevated levels of serum GGT (*t* = 5.024, *P* < 0.0001) and ASAT/ALAT ratio (*P* = 0.008) compared to HC (HC *N* = 16 and AUD patients *N* = 25). (**D**) Fibrometer^®^ parameters showed liver fibrosis only in AUD patients (Fibrosis score: *P* = 0.0099; fibrosis area: *P* = 0.0003; HC *N* = 15 and AUD patients *N* = 23). (**E**) AUD patients had significant elevated serum cytokine (TNF and IL8; respectively *P* = 0.02 and *P* = 0.009; HC *N* = 10 and AUD patients *N* = 27) compared to HC. *P < 0.05, **P < 0.01, ***P < 0.001 Mann–Whitney’s test (or *t*-test only for log GGT).

Compared to HC, AUD patients had significantly lower GM volume in frontal and prefrontal areas, insula, lateral, and medial temporal cortices, cingulate and occipital cortices, but also in subcortical regions including the thalamus, putamen, and caudate nuclei, and in the cerebellum (*P* < 0.001, uncorrected, *k* = 60). These results remained significant after correction for multiple comparisons but with smaller cluster sizes [family‐wise error (FWE), *P* < 0.05; Fig. 1A].

##### WM volume

Compared to HC, AUD patients had significantly lower WM volume in the entire corpus callosum (genu, body and splenium), corona radiata, cingulum, thalamus and cerebellum (*P* < 0.001, uncorrected, *k* = 60). These results (except for the cerebellum) remained significant after correction for multiple comparisons but with smaller cluster sizes (FWE, *P* < 0.05; Fig. 1A).

##### WM integrity

Compared to HC, AUD patients had significantly lower FA values in a large set of fibres including the corpus callosum, the anterior corona radiata, the anterior limb of the internal capsule, the cingulum, the middle cerebellar peduncle and the fornix (*P* < 0.001, uncorrected, *k* = 60). These results remained significant after correction for multiple comparisons but with smaller cluster size (FWE, *P* < 0.05; Fig. 1B).

#### Biological and inflammatory profiles of AUD

Compared to HC, AUD patients had liver dysfunction with elevated levels of GGT and higher ASAT/ALAT ratio (*P* = 0.008; Fig. 1C). Fibrometer analysis revealed liver fibrosis in AUD patients with higher fibrosis score and area compared to HC (*P* = 0.01 and *P* = 0.0003, respectively, Fig. 1D). There was no significant correlation between fibrosis (score or area) and alcohol history variables (AUDIT, alcohol misuse, daily alcohol consumption, Cushman score) in AUD patients (data not shown).

AUD patients also had elevated levels of TNF and IL-8 compared to HC (*P* = 0.02 and *P* = 0.009, respectively; Fig. 1E). None of the cytokines correlated with each other in AUD patients. There was no significant correlation between elevated circulating cytokines (IL-8 and TNF) and alcohol-related variables either: AUDIT (*P* = 0.79 and *P* = 0.36), alcohol misuse (*P* = 0.17 and *P* = 0.08), daily alcohol consumption (*P* = 0.38 and *P* = 0.58) and Cushman score (withdrawal severity score; *P* = 0.86 and *P* = 0.53) in AUD patients.

#### Relationships between liver fibrosis and brain structure

**Figure 2:**
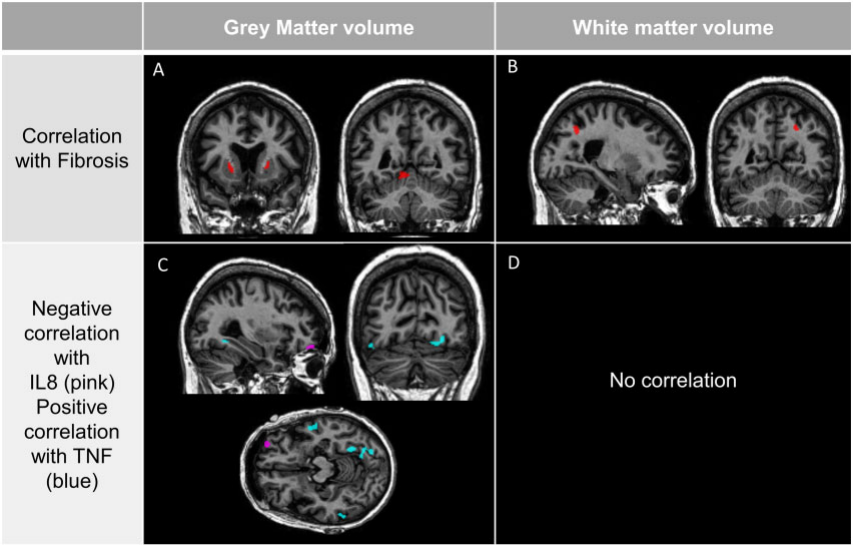
**Correlations between brain alterations, liver fibrosis and inflammation in AUD patients.** In AUD, negative correlations between the fibrosis score and GM volume (**A**), WM volume (**B**) at *P* < 0.001 uncorrected. In AUD, negative correlations between cytokines (negative for IL-8 in purple and positive for TNF in blue) and GM volume (**C**) and an absence of correlation with WM volume (D) at *P* < 0.001. Cluster size: >60 voxels.

Significant negative correlations (*P* < 0.001, uncorrected, *k* = 60) were found between the fibrosis score and GM volume in the putamen (left and right) and cerebellum (lobules 4–5 and the vermis; Fig. 2A). A significant negative correlation (*P* < 0.001, uncorrected, *k* = 60) was also found with the WM volume in the angular gyrus and occipital cortex (Fig. 2B). There was no other significant correlation between liver fibrosis and brain structure.

#### Relationships between circulating cytokines, brain structure and liver function

Given the fact that only the levels of IL-8 and TNF were elevated in AUD compared with HC, we conducted correlations between brain structure and these cytokines only.

A significant negative correlation (*P* < 0.001, uncorrected, *k* = 60) was found between IL-8 levels and GM volume in the orbitofrontal region (Fig. 2C). Significant positive correlations (*P* < 0.001, uncorrected, *k* = 60) were found between TNF levels and GM volume in the prefrontal and temporal cortices and in the cerebellum (Fig 2C). There was no other significant correlation between these cytokines and brain structure.

We found a positive correlation between IL-8 levels on the one hand and fibrosis score (*r* = 0.64; *P* = 0.006) and fibrosis area (*r* = 0.73; *P* < 0.001; data not shown) on the other hand.

### Preclinical data

#### Alcohol consumption

Over the 50-week period, weekly alcohol intake in alcohol-drinking rats averaged between 35 and 50 g/kg (Supplementary Fig. 2). These weekly intakes were suggestive of daily alcohol intakes of 5.0–7.5 g/kg, i.e. the standard daily amount of alcohol voluntarily consumed by male sP rats when exposed to the homecage 2-bottle ‘alcohol *vs* water’ choice regimen with unlimited access.^43^ Weekly mean alcohol preference (defined as the ratio between the amounts of alcohol solution consumed and the total liquid intake) rose from 65% (Week 1) to 95% (from Week 7 onwards) (data not shown), reproducing a basal feature of alcohol drinking in sP rats.^43^

#### Impact of 50-week alcohol drinking on brain structure

**Figure 3:**
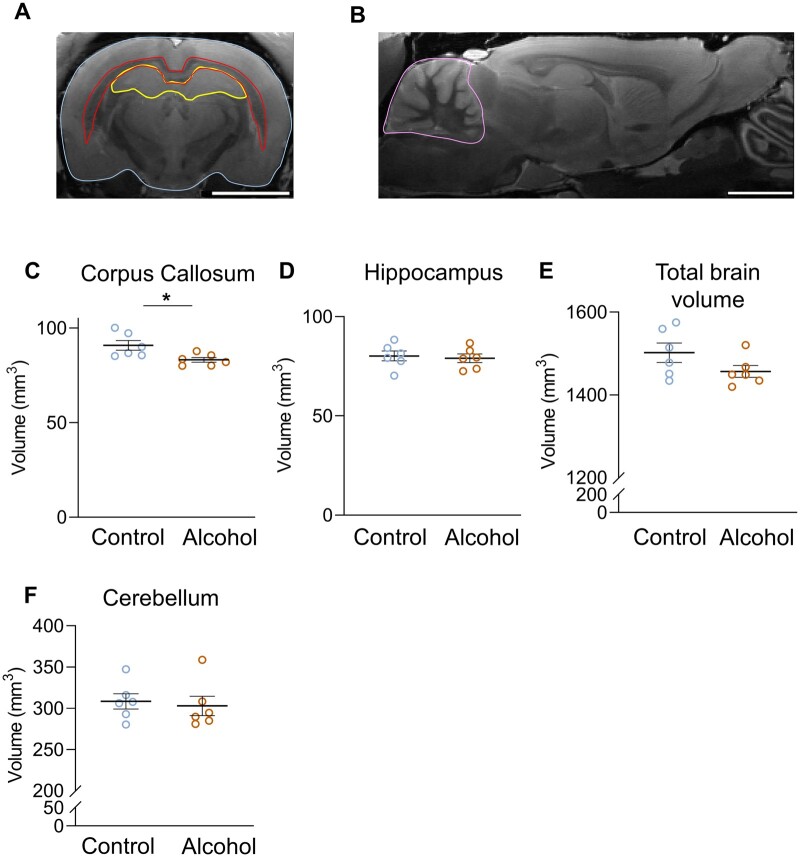
**Chronic alcohol consumption specifically reduces the volume of the corpus callosum in sP rats.** (**A**) Representative scan of brain MRI (coronal view) with total brain, corpus callosum and hippocampus (delimited respectively in blue, red, and yellow). (**B**) Representative scan of brain MRI (sagittal view) used to measure cerebellum volume (delimited in pink). Total volume of the corpus callosum (**C**), hippocampus (**D**), total brain (**E**) and cerebellum (**F**) in control and alcohol-drinking rats, respectively *P* = 0.026, *P* = 0.081, *P* = 0.179, *P* = 0.699. *P < 0.05, Mann–Whitney’s test, *N* = 6.

The entire corpus callosum (genu, body, splenium) had significantly smaller volume in alcohol-drinking rats compared to control rats (*P* = 0.031; Fig. 3C). There was no difference of volume in other brain regions (hippocampus, cerebellum, and total brain volume; Fig. 3D–F).

**Figure 4:**
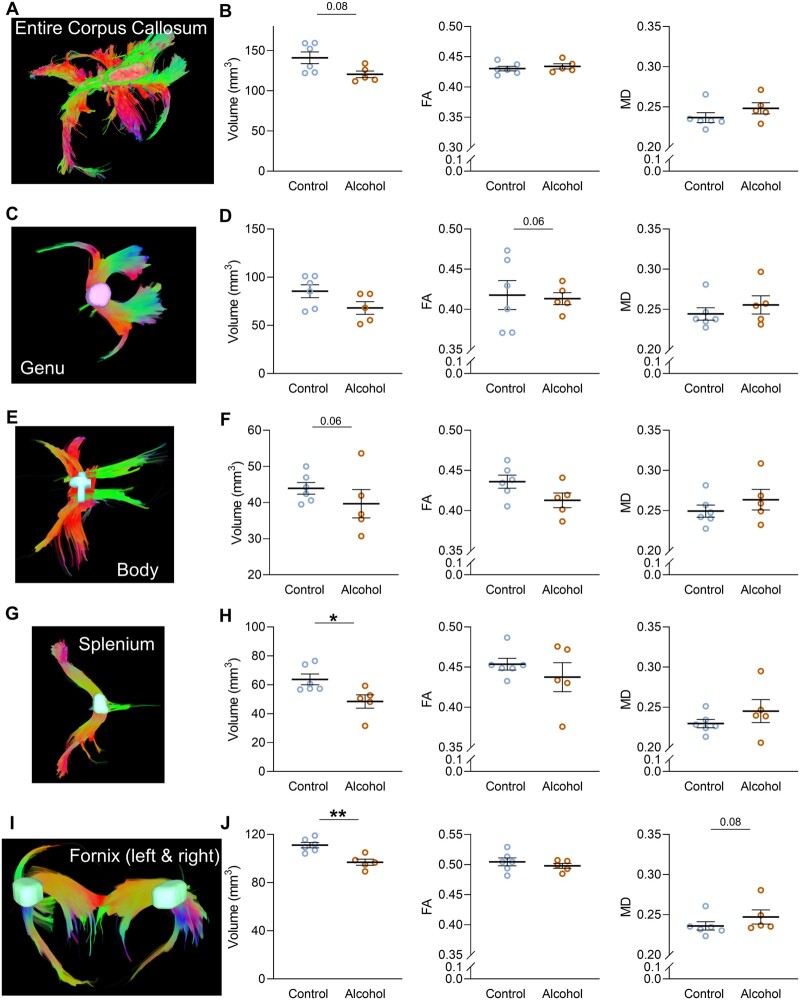
**Chronic alcohol drinking impairs both corpus callosum volume and fibre integrity in sP rats.** (**A**) Individual reconstruction of fibre tracts in the entire corpus callosum. (**B**) White matter (WM) microstructure parameters in the entire corpus callosum of control and alcohol-drinking sP rats (Volume *P* = 0.08; FA *P* = 0.33; MD *P* = 0.12). (**C**) Individual reconstruction of fibre tracts in the genu of the corpus callosum. (**D**) WM microstructure parameters in the genu of the corpus callosum of control and alcohol-drinking sP rats (Volume *P* = 0.24; FA *P* = 0.06; MD *P* = 0.33). (**E**) Individual reconstruction of fibre tracts in the body of the corpus callosum. (**F**) WM microstructure parameters in the body of the corpus callosum of control and alcohol-drinking sP rats (Volume *P* = 0.06; FA *P* = 0.5; MD *P* = 0.21). (**G**) Individual reconstruction of fibre tracts in the splenium of the corpus callosum. (**H**) WM microstructure parameters in the splenium of the corpus callosum of control and alcohol-drinking sP rats (Volume *P* = 0.01; FA *P* = 0.29; MD *P* = 0.16). (**I**) Individual reconstruction of fibre tracts in the entire fornix (left and right). (**J**) WM microstructure parameters in the entire fornix of control and alcohol-drinking sP rats (Volume *P* = 0.004; FA *P* = 0.33; MD *P* = 0.08). *P < 0.05, **P < 0.01; control *N* = 6, alcohol-drinking *N* = 5; Mann–Whitney test.

Region-based tractography revealed microstructural alterations of the corpus callosum and fornix in alcohol-drinking rats compared to control rats. Fibres volume of the entire corpus callosum and more precisely in the body and splenium was significantly lower in alcohol-drinking rats compared to control rats (Fig. 4B, F, G). FA in the genu of the corpus callosum was decreased in alcohol-drinking rats, although the difference did not reach statistical significance (*P* = 0.06; Fig. 4D). In the fornix, the volume of fibres was significantly lower in alcohol-drinking rats (*P* = 0.004) and there was a trend for a higher MD (*P* = 0.08; Fig. 4J).

#### Impact of 50-week alcohol drinking on brain inflammation

**Figure 5:**
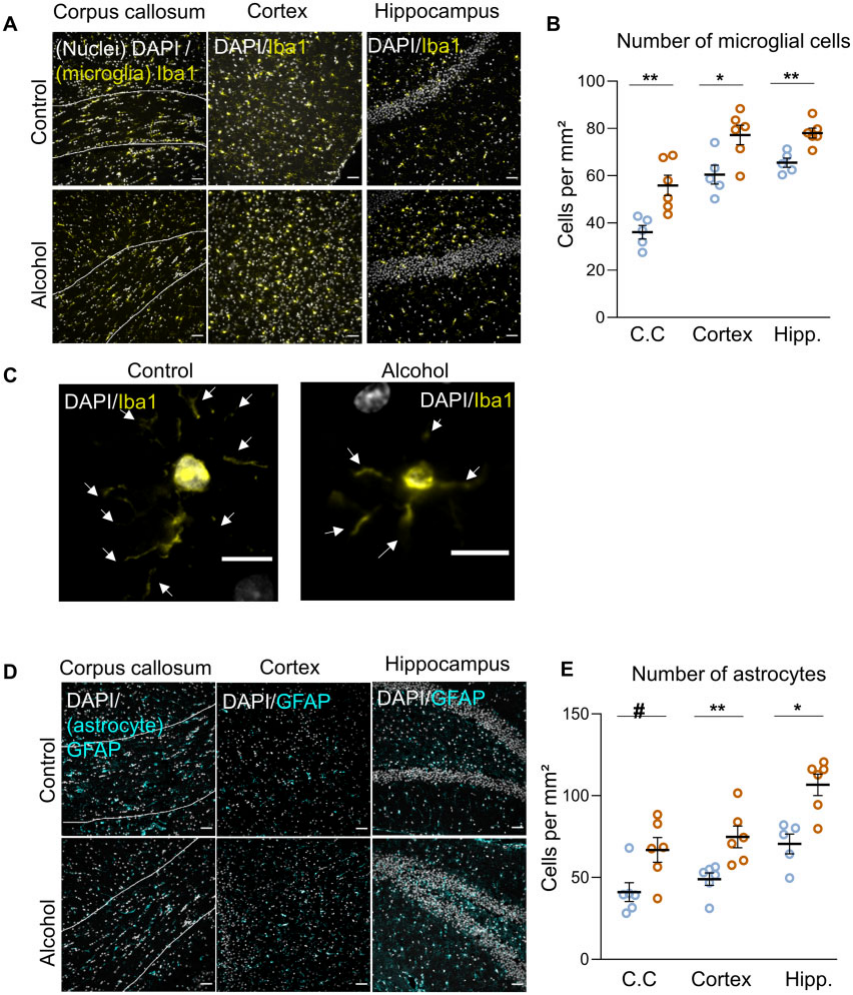
**Neuroinflammatory responses to chronic alcohol drinking in sP rats: microgliosis and astrogliosis in the corpus callosum, brain cortex and hippocampus, in the absence of neuronal death.** (**A**) Representative photomicrographs of microglia (Iba1+ cells) in the corpus callosum (delimited with dotted line), cortex and hippocampus in control and alcohol-drinking sP rats. Scale bar 50 µm. (**B**) Quantification of microglial density in the corpus callosum (*P* = 0.004), the cortex (*P* = 0.03) and the hippocampus (*P* = 0.0065). (**C**) Representative photomicrographs of microglial cell morphology in control and alcohol-drinking sP rats. Note the decreased process density (arrows) in alcohol-drinking rats. Scale bar 10 µm. (**D**) Representative photomicrographs of astrocytes (GFAP+ cells) in the corpus callosum (delimited with dotted line), cortex and hippocampus of control and alcohol-drinking sP rats. Scale bar 50 µm. (**E**) Quantification of astrocytic density in the corpus callosum (*P* = 0.065), the cortex (*P* = 0.004) and the hippocampus (*P* = 0.017). Scale bar 100 µm #*P* < 0.1, *P < 0.05, **P < 0.01, control *N* = 6, alcohol-drinking *N* = 6, Mann–Whitney test.

We measured microglial (Iba1^+^ staining, Fig. 5A) and astrocytic (GFAP^+^ staining, Fig. 5D) density in the corpus callosum, cortex and hippocampus by immunohistochemistry. The density of microglial cells (Iba1^+^ cells) was significantly higher in alcohol-drinking rats in all these regions (Fig. 5B). The number of GFAP^+^ cells was also higher in alcohol-drinking rats. In the cortex and the hippocampus, the difference was statistically significant (*P* < 0.05; Fig. 5E); only a trend was found in the corpus callosum (*P* < 0.1; Fig. 5E).

Fluorojade C staining and caspase 3 did not reveal any neurodegeneration and apoptosis in alcohol-drinking and control rats (data not shown).

#### Impact of 50-week alcohol drinking on liver inflammation

**Figure 6:**
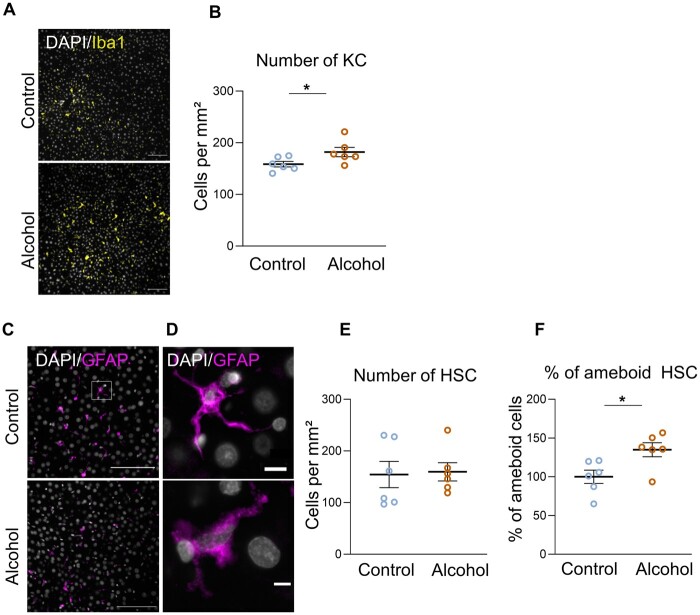
**Chronic alcohol drinking provokes liver inflammation in sP rats.** (**A**) Representative photomicrographs of Kuppfer cells (Iba1+) in the liver of control and alcohol-drinking sP rats. Scale bar 100 µm. (**B**) Quantification of Kuppfer cell density (*P* = 0.036). (**C**) Representative photomicrograph of hepatic stellate cells (HSC) (GFAP+) in the liver of control and alcohol-drinking sP rats. Scale bar 100 µm. (**D**) Detail of HSC. Note the amoeboid shape in the alcohol-drinking condition, characteristic of the activated state of HSC. Scale bar 10 µm. (**E**) Quantification of HSC density (*P* = 0.59). (**F**) Percentage of amoeboid HSC in the liver of alcohol-drinking and control sP rats (*P* = 0.026). *P < 0.05, control *N* = 6, alcohol-drinking *N* = 6 Mann–Whitney test.

We measured the number of Kupffer cells (KC) (resident macrophages in the liver) (Iba 1^+^ cells; Fig. 6A) and of hepatic stellate cells (HSC) (GFAP^+^ cells; Fig. 6C). We found a higher number of KC in the liver of alcohol-drinking rats compared to control rats (*P* < 0.05; Fig. 6B).

The number of HSC (main effectors of liver fibrosis) was similar in both groups (Fig. 6E). However, the morphology of HSC was different in the two conditions: control rats showed ramified HSC cells (corresponding to a quiescent phenotype), whereas alcohol-drinking rats showed more amoeboid HSC, suggestive of activated HSC (Fig. 6D and F). When cells were ramified there was no difference in the number of ramifications per cell (data not shown).

Hematoxylin & Eosin staining revealed no major difference in the liver structure and indicated an absence of fibrosis, steatosis or neutrophil infiltration (Supplementary Fig. 1).

## Discussion

The objective of the present study was to gain a better understanding of the contribution of inflammation and liver fibrosis to alcohol-related brain damage. The main novelty was to use a translational approach including a biological and neuroimaging investigation of AUD patients as well as an *ex-vivo* imaging and immunohistological examination of sP rats.

### Brain and liver abnormalities, and systemic inflammation in AUD patients

In accordance with previous studies, we showed that AUD patients had macrostructural brain abnormalities in both GM and WM cortical and subcortical regions.^8^^,^^10^ We also found widespread microstructural alterations in AUD patients consistent with recent investigations.^10^^,^^15^ Even in the absence of cirrhosis, AUD patients had alcohol-related liver dysfunction.^22^^,^^46^

Regarding inflammation, we did not find any difference in the circulating levels of the proinflammatory cytokines monocyte chemoattractant protein-1 and macrophage inflammatory protein 1β between controls and AUD patients, in contrast to previous studies.^47–50^ It is worthwhile mentioning that these previous studies were conducted in patients with alcoholic advanced liver disease, hepatitis or non-alcoholic steatohepatitis, whereas patients included in the present study did not exhibit any ostensible clinical signs of liver disease. Despite the fact that previous studies have found that monocyte chemoattractant protein-1 could be a biomarker of liver disease, our data revealed that this chemokine is not related to early or discrete liver dysfunction.

In accordance with previous studies,^21^^,^^51^ we found higher plasmatic levels of the pro-inflammatory cytokines IL-8 and TNF in AUD patients compared to HC. Serum TNF concentration was reported to be higher in AUD patients than in the general population, regardless of the level of alcohol consumption^52^^,^^53^ and to decrease within the first days of abstinence.^54^^,^^55^ By contrast, IL-8, which is not primarily associated with acute inflammation and rather acts as a potential angiogenic factor and chemotactic attractor for all known types of migratory immune cells, remains increased at least for the first 6 months after alcohol withdrawal.^54^

### Liver impairment and systemic inflammation are associated with regional brain volume

While systemic inflammation is known to be associated with cognitive impairments and cerebral damage in cirrhotic patients in the case of encephalic encephalopathy, studies of these relationships in non-cirrhotic patients are scarce. Some findings suggested that systemic inflammation and liver fibrosis correlate with behavioural impairments in non-cirrhotic patients^21^^,^^22^^,^^56^ but, to our knowledge, there is no study on the potential relationship between systemic inflammation and structural brain damage.

The present results showed that circulating proinflammatory cytokines and liver fibrosis correlated with brain macrostructure in recently detoxified AUD patients without ostensible liver damage. We found a specific vulnerability of putamen to liver fibrosis. Accordingly, the basal ganglia volume, including the putamen, is altered in severe liver disease.^57^ Liver function has also been related to brain atrophy.^58^ However, a major limitation of that previous study was that liver function was only assessed with GGT and transaminases levels, which are not specific to liver fibrosis or alcohol-related liver damage. In addition to a clear relationship between liver function and specific regional brain volume, we show here that levels of circulating proinflammatory cytokines were associated with brain volumes. Positive and negative immune correlations have also been found in other neurological diseases such as schizophrenia^59–61^ or Alzheimer’s disease^62^ indicating neuroinflammation with microglial activation. IL-8 and TNF levels correlate with GM volume in regions typically impaired in AUD: prefrontal cortex (with orbitofrontal cortex), temporal cortex and cerebellum. Proinflammatory cytokines could come from the liver^63^ and in particular from activated KC. One possible mechanistic hypothesis is that alcohol could directly (or indirectly) activate KC, which would initiate the synthesis of proinflammatory cytokines that would be released to the bloodstream and reach the brain.

In a previous study, chronic alcohol drinking induced increased levels of LPS in the serum of sP rats and altered their intestinal microbiota composition.^64^ These results have also been shown in alcohol non-preferring rats and AUD patients.^33^ Several studies have shown that increased LPS circulating levels induced by alcohol stimulate KC to generate ROS and cytokines.^65^ These inflammatory mediators subsequently activate HSCs via a Toll-like receptor 4 (TLR4) signalling pathway, which eventually results in enhanced, chronic production of extracellular matrix proteins—and promotion of fibrogenesis.^66^^,^^67^ Additionally, HSCs are also enriched with TLR4 to which LPS directly binds, and can thus activate through LPS signalling.^68^

### Chronic alcohol exposure induced similar brain abnormalities in a rat model of AUD

sP rats have been selectively bred for high alcohol preference and consumption. Notably, sP rats meet all the fundamental requirements posed when defining an animal model of AUD, including tolerance and behavioural dependence.^43^^,^^69^ The results collected in sP rats were similar to those observed in AUD patients in terms of macrostructural and microstructural brain alterations, in line with previous preclinical investigations.^15^^,^^19^ Chronic (50 consecutive weeks) alcohol drinking resulted in a lower WM volume in the fornix and corpus callosum, potentially reflecting myelin and/or axonal damage or glial/cellular reaction during neuroinflammation phenomenon.^15^

It should be noted that the water diffusivity is reduced *ex-vivo* and that the diffusion images obtained in MRI cannot be compared directly between the AUD Patients and the sP rats. To overcome this issue, we do not compare the raw diffusion values but the patterns of brain abnormalities in the different groups.

### Chronic alcohol drinking induces brain and liver inflammatory responses

We describe here, for the first time, an alcohol-induced inflammatory response in the brain of sP rats after 50 consecutive weeks of voluntary alcohol drinking. Importantly, this inflammatory response was not accompanied by signs of neurodegeneration or apoptosis. Chronic alcohol drinking induces innate immune signalling cascades through the activation of the proinflammatory transcription factor, NF-κB.^70–72^ Post-mortem human AUD brain has been found to have increases in microglial markers^73^ as well as increased expression of IL-8.^74^ We found a significant higher number in microglia and astrocytes in all the brain regions studied (cortex, corpus callosum and hippocampus). In addition, microglial cells in alcohol-drinking rats showed morphological alterations characterized by a transition from a resting and ramified morphology, to an amoeboid phenotype compatible with a partially activated state. Several studies have proposed that partially activated microglia is necessary in neuroprotection and axonal regeneration,^75^^,^^76^ an assumption compatible with the absence of neurodegeneration observed in the present study.

Fifty weeks of alcohol drinking provoked a series of inflammatory responses in the liver of sP rats, including KC proliferation and HSC activation. These responses were not accompanied by clear signs of liver damage (no significant steatosis, fibrosis, or neutrophil infiltration). These results indicate that chronic alcohol consumption induces inflammatory responses in the liver even in absence of hepatitis or cirrhosis, with activation of HSC. These cells, in addition of playing a role in the development of steatosis and fibrosis, are major producers of cytokines and chemokines that are released to the bloodstream. Chronic alcohol consumption may thus directly provoke inflammatory responses in the brain and indirectly through the trigger of peripheral cytokines reaching the brain. In agreement with this hypothesis, we found a correlation between peripheral cytokine levels and brain volumes in AUD patients as well as microglial activation in a rodent model of AUD.

### Limitations, methodological considerations and future perspectives

The similarities in the results concerning WM abnormalities in the AUD patients and chronic alcohol-drinking rats are one of the major findings of this study. However, it should be noted that we have analysed in vivo samples in human and ex vivo samples in rats, and that there are significant basal differences in water diffusion and cellular death. Recent studies have shown that ex vivo use of diffusion MRI are comparable with human and preclinical models *in vivo*.^77^^,^^78^ Ideally, tract-based spatial statistics (TBSS) would have been the method of choice to analyse both the animal and human datasets. However, for the animal dataset, the brain extraction, segmentation and warping algorithms available did not provide a reliable result, making a voxel-based analysis impossible. The best and most robust option available was thus through manual delineation. The dataset in humans has already been analysed with TBSS before.^40^ Compared with TBSS, significant clusters from voxel-based morphometry studies are mathematically ‘closer’ to ROI measurements, as they are both a reflection of the average value within that cluster. On the contrary, TBSS measurements come from a skeletonized dataset where data across a tract are effectively one-voxel thick and, numerically, take the maximum value across the projection perpendicular to the local skeleton structure for each voxel within the skeleton.^79^ Hence, we preferred a voxel-based non-TBSS approach in our analyses for the human dataset.

One major limitation of this study is the absence of information about cytokines levels in the serum or in the brain of sP rats. A recent study using sP rats of the same cohort used in the present study found similar levels of TNF in the serum of control and alcohol-drinking rats,^64^ which would suggest an independent effect of alcohol on the brain and liver in terms of inflammatory responses.

Also, other factors, such as genetics, dietary status, gut permeability and the intestinal biome, could play a role on liver inflammation and the development of neuroinflammation and neurodegeneration.^56^^,^^80^ It has been shown that, in sP rats, the expression of neuropeptides and microglia activation was altered in several brain regions compared to the alcohol-nonpreferring counterpart, indicating pre-existing genetic differences in alcohol-induced responses.^81^

Our results show that alcohol provokes liver and brain inflammatory responses that correlate with brain abnormalities even in the absence of advanced liver damage in both AUD patients and alcohol-preferring sP rats consuming alcohol chronically. Future studies are needed to elucidate causality links regarding the brain–liver interactions (i.e. whether alcohol-induced liver and brain inflammatory responses arise independently or whether liver and brain inflammatory responses are related), in order to find new therapeutic strategies aiming at blocking alcohol-related brain damage.

## Supplementary material

Supplementary material is available at *Brain Communications* online.

## Competing interests

The authors report no competing interests.

## Supplementary Material

fcab154_Supplementary_Data

## Abbreviations

ALAT =: alanine transaminase
ASAT =: aspartate transaminase
AUD =: alcohol use disorder
DSM-5 =: Diagnostic and Statistical Manual of Mental Disorders, 5th edition
DTI =: diffusion tensor imaging
FA =: fractional anisotropy
GFAP =: glial fibrillary acidic protein
GGT =: gamma-glutamyl transferase
GM =: grey matter
HCs =: healthy controls
HE =: hepatic encephalopathy
HSC =: hepatic stellate cells
IL8 =: interleukin 8
KC =: Kupffer cells
sP rats =: sardinian alcohol-preferring rats
TBSS =: tract-based spatial statistics
TNF =: tumour necrosis factor
WM =: white matter
