# Supplementary material for: Role of inflammation in alcohol-related brain abnormalities: a translational study
Source: Brain Commun. 2021 Jul 16;3(3):fcab154. doi: 10.1093/braincomms/fcab154 (PMC8361421; doi:10.1093/braincomms/fcab154)
Supplement: fcab154_Supplementary_Data [file fcab154_supplementary_data.zip › Original submission.pdf]

OXFORD UNIVERSITY PRESS | Brain Communications

Role of inflammation in alcohol-related brain abnormalities: a translational study

Journal:	*Brain Communications*
Manuscript ID	BRAINCOM-2021-016
Manuscript Type:	Original Article
Date Submitted by the Author:	12-Jan-2021
Complete List of Authors:	Lanquetin, Anastasia; INSERM U1237,<br>Leclercq, Sophie; Institute of Neuroscience and Louvain Drug Research Institute , UCLouvain, Université Catholique de Louvain<br>de Timary, Philippe; Institute of Neuroscience and Louvain Drug Research Institute , Université Catholique de Louvain<br>Segobin, Shailendra; Inserm , U1077<br>Naveau, Mikaël; Normandie Univ UNICAEN, CNRS, UMS 3408, GIP Cyceron<br>Coulbault, Laurent ; Centre Hospitalier Universitaire, Caen, Service de Biochimie<br>Maccioni, Paola; Neuroscience Institute, Section of Cagliari, National Research Council of Italy<br>Lorrai, Irene; Neuroscience Institute, Section of Cagliari, National Research Council of Italy<br>Colombo, Giancarlo; Neuroscience Institute, Section of Cagliari, National Research Council of Italy<br>Vivien, Denis; INSERM U1237<br>Rubio, Marina; INSERM U1237<br>Pitel, Anne Lise; Inserm U923,
Keywords:	

SCHOLARONE™
Manuscripts

Role of inflammation in alcohol-related brain abnormalities: a translational study

Authors: Anastasia Lanquetin[1], Sophie Leclercq[2], Philippe de Timary[2], Shailendra Segobin[3], Mikaël Naveau[4], Laurent Coulbault[5], Paola Maccioni[6], Irene Lorrai[6], Giancarlo Colombo[6], Denis Vivien[1,7], Marina Rubio[1]*, Anne-Lise Pitel[3,8]*

*These authors contributed equally

Affiliations:

(1) Normandie Univ, UNICAEN, INSERM, PhIND "Physiopathology and Imaging of Neurological Disorders", Institut Blood and Brain @ Caen-Normandie, Cyceron, 14000 Caen, France

(2) Institute of Neuroscience and Louvain Drug Research Institute, UCLouvain, Université Catholique de Louvain, Brussels, Belgium

(3) Normandie Univ, UNICAEN, PSL Université Paris, EPHE, INSERM, U1077, CHU de Caen, GIP Cyceron, Neuropsychologie et Imagerie de la Mémoire Humaine, 14000 Caen, France

(4) Normandie Univ UNICAEN, CNRS, UMS 3408, GIP Cyceron, Caen, France

(5) Caen University Hospital, Biochemistry Department, Normandie University, UNICAEN, EA 4650, Caen, France

(6) Neuroscience Institute, Section of Cagliari, National Research Council of Italy, 09042 Monserrato (CA), Italy

(7) Department of Clinical Research, CHU Côte de Nacre, Caen, France

(8) Institut Universitaire de France (IUF)

Running title: Alcohol-related inflammation

For Review Only

Abstract

Brain abnormalities observed in AUD (alcohol use disorder) are highly heterogeneous in nature and severity, possibly because chronic alcohol consumption also affects peripheral organs leading to comorbidities that can result in exacerbated brain alterations. Despite numerous studies focusing on the effects of alcohol on the brain or liver, few studies have simultaneously examined liver function and brain damage in AUD, and even fewer investigated the relationship between them except in hepatic encephalopathy. And yet, liver dysfunction may be a risk factor for the development of alcohol-related neuropsychological deficits and brain damage well before the development of liver cirrhosis, and potentially through inflammatory responses. The use of animal models enables a better understanding of the pathophysiological mechanisms underlying liver-brain relationships in AUD, and more particularly of the inflammatory response at the tissue, cerebral, and hepatic levels.

The objective of this translational study was to investigate, both in AUD patients and in a validated animal model of AUD, the links between peripheral inflammation, liver damage, and brain alterations. To do this, we conducted an *in vivo* neuroimaging examination and biological measures to evaluate brain volumes, liver fibrosis, and peripheral cytokines in AUD patients. In selectively bred Sardinian alcohol-preferring (sP) rats, we carried out *ex vivo* neuroimaging examination and immunohistochemistry to evaluate brain and liver inflammatory responses after chronic (50 consecutive weeks) alcohol drinking.

In recently abstinent and non-cirrhotic AUD patients, the score of liver fibrosis positively correlated with subcortical regions volumes (especially in right and left putamen) and level of circulating proinflammatory cytokines. In sP rats, we found macrostructural brain damage and microstructural white matter abnormalities similar to those found in AUD patients. In addition,

For Review Only

in agreement with the results of peripheral inflammation observed in the patients, we revealed, in sP rats, inflammatory responses in the brain and liver caused by chronic alcohol consumption.

Since the liver is the main source of cytokines in the human body, these results suggest a relationship between liver dysfunction and brain damage in AUD patients, even in the absence of major liver disease. These findings encourage considering new therapeutic strategies aiming at treating peripheral organs to limit alcohol-related brain damage.

Keywords: alcohol use disorders; inflammation; brain; MRI

For Review Only

Introduction

Alcohol use disorder (AUD) is a chronic relapsing brain disease, which represents a major public health problem affecting millions of people around the world (Rehm *et al.*, 2015). The most recent version of the Diagnostic and Statistical Manual of Mental Disorders (DSM-5, American Psychiatric Association, 2013) no longer considers AUD as a categorical disease (abuse versus dependence, as in previous version, DSM-IV, American Psychiatric Association, 1994) but offers a dimensional approach with a spectrum of severity ranging from mild, moderate, to severe AUD. DSM-5 also acknowledges alcohol-related neurocognitive disorders in agreement with the literature that frequently reports altered brain structure and neuropsychological impairments in AUD patients (Chanraud *et al.*, 2009; Pfefferbaum *et al.*, 2009; Pitel *et al.*, 2015).

Chronic alcohol consumption is indeed associated with brain alterations that can be revealed *in vivo* by magnetic resonance imaging. In AUD patients, gray matter (GM) structural abnormalities can affect cortical and subcortical regions. The frontal cortex seems to be especially sensitive to chronic and heavy alcohol consumption (Mackey *et al.*, 2019), but shrinkage has also been observed in temporal, parieto-occipital cortex and in the cerebellum (Pitel *et al.*, 2012). In subcortical regions, atrophy of the hippocampus (Wilson *et al.*, 2017), mammillary bodies, cingulate cortex and thalami (Pitel *et al.*, 2012; Segobin *et al.*, 2019) have also been reported.

Regarding white matter (WM), MRI studies revealed a thinning of the corpus callosum (Pfefferbaum *et al.*, 1996; Pitel *et al.*, 2010). Diffusion tensor imaging (DTI) and in particular the measure of fractional anisotropy (FA), are specific MRI techniques that explore noninvasively WM microstructure and integrity (myelination or axonal integrity; Harper & Kril, 1988; Koike *et al.*, 2001). DTI studies conducted in AUD patients have shown

For Review Only

microstructural alterations in the fornix and corpus callosum (Pfefferbaum and Sullivan, 2005; Pfefferbaum *et al.*, 2009; De Santis *et al.*, 2019), even when no structural MRI abnormalities were observed (Pfefferbaum *et al.*, 2000; Pfefferbaum and Sullivan, 2002). While structural brain abnormalities are currently relatively well characterized in AUD patients, the pathophysiology underlying these brain damages remain unclear and need being explored, in particular by using animal models.

Preclinical neuroimaging studies suggested that chronic alcohol drinking resulted in ventricular expansion, hippocampus shrinkage (Pfefferbaum *et al.*, 2006*a*; Frischknecht *et al.*, 2017), and decrease in volume and FA in fiber tracts of the corpus callosum and fornix (De Santis *et al.*, 2019) in rats.

Our groups are interested in testing the possibility that damages developed at the level of peripheral organs participate to the development of the brain (Leclercq *et al.*, 2014; Ritz *et al.*, 2016). Hepatic encephalopathy (HE) induced by chronic alcohol consumption is the extreme example of brain and liver interaction. However, long before the development of a full-blown HE, altered liver function may affect brain structure and function. Ritz et al. (2016) for instance observed that liver fibrosis is related and may partially explain executive dysfunction in AUD patients without clinically detectable HE. Junghanns et al. (2004) also observed that GGT levels were related to mental flexibility abilities. These results indicate that liver dysfunction may predict the severity of executive impairments in AUD patients, suggesting the existence of a liver brain communication. Peripheral inflammation could be one of these pathways of communication, where the release of cytokines could contribute to neuroinflammation (Leclercq *et al.*, 2014; D'Mello and Swain, 2017).

Preclinical and human *post-mortem* studies have suggested neuroinflammation as an important pathogenic process participating to alcohol-induced brain damage (Qin *et al.*, 2008), with a pivotal role of microglial activation (Zhao *et al.*, 2013; Alfonso-Loeches *et al.*, 2016;

For Review Only

Montesinos *et al.*, 2016). However, the study of neuroinflammation in AUD patients by positron emission tomography (PET) using TSPO ligand (used to evaluate microglial activation *in vivo*) has shown controversial results, with either lower or equal levels of neuroinflammation in AUD patients compared to controls (Kalk *et al.*, 2017; Kim *et al.*, 2018; Tyler *et al.*, 2019). Circulating cytokines can be used to evaluate the inflammatory state in AUD patients. Cytokines are considered important mediators of the systemic-brain communication, as they can reach the central nervous system and induce neuroinflammation that is associated with changes in mood, cognition, and drinking behavior (Leclercq *et al.*, 2017 for review).

The objectives of this translational study were to investigate, both in AUD patients and in selectively bred alcohol-preferring rats, the links between peripheral inflammation, liver damage and brain alterations. To do so, we have examined grey and white brain matter abnormalities, liver damage, and peripheral cytokines levels in a cohort of recently detoxified AUD patients as well as in chronically alcohol drinking Sardinian alcohol-preferring (sP) rats.

Our results show that, in both the human and the animal model, alcohol consumption provokes liver and brain inflammatory responses that are correlated with brain abnormalities, in the absence of advanced liver damages.

For Review Only

Material and methods

Clinical data

Participants

Forty-one participants were included in this study: 25 patients with AUD and 16 healthy controls (HC). None of them had a history of neurological pathology, endocrinal nor infectious diseases, depression (assessed using the Beck Depression Inventory [BDI]; Beck *et al.*, 1961) nor other forms of substance use disorder (except tobacco). All participants were informed about the study approved by the local ethics committee of Caen University Hospital (CPP Nord Ouest III, no. IDRCB: 2011-A00495-36) prior to their inclusion and provided their written informed consent. The study was carried out in line with the declaration of Helsinki (1964).

For Review Only

Clinicians recruited AUD patients while they were receiving withdrawal treatment as inpatients at Caen University Hospital. AUD patients met “alcohol dependence” criteria according to the DSM-IV-TR (American Psychiatric Association., 1994) and “severe AUD” criteria according to the DSM-5 (American Psychological Association (APA), 2013) for at least 5 years. At inclusion and evaluation, none of them presented physical symptoms of alcohol withdrawal as assessed by Cushman's scale (score<= 2; Cushman *et al.*, 1985) nor were under medication by benzodiazepines. Alcohol history of the AUD patients is described in Table 1.

HC were recruited locally and to match the demographics of the AUD patients. They were interviewed with the Alcohol Use Disorders Identification Test (AUDIT) to ensure that they did not meet the criteria for alcohol abuse (AUDIT < 7 for men and <6 for women; Gache *et al.*, 2005). None of the controls had a BDI score > 29 and a score <136 on the Dementia Rating Scale (DRS-2; Jurica *et al.*, 2001).

AUD patients and HC were age-, sex-, and education-matched (Table 1).

Biological assessment

Fasting blood samples were collected for all participants, either at inclusion (HC) or the day after admission to hospital (AUD) (24h) to ensure that patients were free from alcohol.

Liver function was assessed using the level of GGT and the ASAT/ALAT ratio (a ratio > 1 suggested alcoholic liver disease (Halvorson *et al.*, 1993)). We used the FibroMeter® for the non-invasive diagnosis of alcoholic liver fibrosis based on the measure of 4 blood markers: alpha 2-macroglobulin, platelets, prothrombin ratio and hyaluronic acid (Calès *et al.*, 2005). This yielded a fibrosis score (ranging from 0 to 1) and a percentage of fibrosis area (ranging from 0 to 100%).

Serum levels of the four inflammatory cytokines IL-8, TNF, MCP-1 and MIP-1β were assayed by a multiplex cytokine assay (Human Bio-Plex; Bio-Rad Laboratories Inc., Hercules, CA, USA) according to instructions from the manufacturer.

Magnetic Resonance Imaging Data Acquisition

Brain imaging examinations were conducted in 16 HC and 25 AUD patients.

A high-resolution T1-weighted anatomical image was acquired for each subject on a Philips Achieva 3T scanner (Philips Healthcare/Philips Medical Systems International B.V., Eindhoven, the Netherlands) using a 3-dimensional fast-field echo sequence (sagittal; repetition time, 20 ms; echo time, 4.6 ms; flip angle, 10°; 180 slices; slice thickness: 1 mm; field of view, 256 × 256 mm2; matrix, 256 × 256).

Regarding diffusion tensor imaging (DTI), 70 slices (thickness: 2 mm, no gap) were acquired axially using a diffusion-weighted imaging spin echo sequence (32 directions at b = 1,000 s/mm2, repetition time = 10,000 ms, echo time = 82 ms, flip angle = 90°, field of view = 224 ×

For Review Only

224 mm2, matrix = 112 × 112, and in-plane resolution = 2 × 2 mm2; 1 no–diffusion-weighted image [DWI] at b = 0 s/mm2 was also acquired).

Magnetic Resonance Imaging Data Processing

The volumetric MRI data of gray and white matter were analyzed using the Statistical Parametric Mapping software (SPM12; Wellcome Department of Cognitive Neurology, Institute of Neurology, London, UK). Pre-processing steps included segmentation of the MRI data into GM, WM and CSF, and spatial normalization to the Montreal Neurological Institute (MNI) template (voxel size = 1.5 mm^3, matrix = 121 × 145 × 121). The resulting images were smoothed by a Gaussian kernel of 8 mm full width at half maximum (FWHM). GM (and WM) measures reflect cerebral macrostructure and numerically corresponds to the mean GM (or WM) signal per unit volume for each significant cluster.

The DTI data were processed as previously described by Segobin et al. (2015) to create FA maps. Numerically, FA values vary between 0 and 1. Generally, the higher the FA value, the better the microstructural integrity of the fiber within that voxel. FA is assumed to be a structural biomarker that depicts white matter (WM) disruption involving myelin, cytoskeleton, and the axons' microtubule system (Pfefferbaum *et al.*, 2006*b*).

A GM mask was obtained taking the unmodulated GM images of HC normalized to the MNI space, averaging them, and thresholding the resultant mean image at 0.5. We obtained a WM macrostructure mask with the same procedure. The WM microstructural integrity mask was obtained by taking the FA maps of HC normalized to the MNI space, averaging them, and thresholding the resultant mean image at 0.3. The resulting GM and WM masks were applied, respectively, to GM and WM volumes and WM microstructural integrity data analyses.

Statistical analysis

Biological assessment of the AUD patients

For Review Only

The normality of the distribution of the laboratory measures for the AUDand HC groups was examined using the Shapiro-Wilk test. Mann-Whitney tests were then used to compare the two groups for measures of liver function and inflammatory cytokines.

Pattern of Brain Alterations and Correlations with biological variables

Voxel-based t-tests were conducted in SPM12 to compare HC and AUD on GM volume, WM volume and WM integrity (FA values). Results are reported at $p < 0.05$ (FWE-corrected) and at $p < 0.001$ (uncorrected for multiple comparisons) with a minimal cluster size (k) of 60 voxels (200 mm3 for volumetric analysis; 60mm3 for DTI analysis)

Voxel-based multiple linear regressions were then conducted in SPM12 to examine, only in AUD patients, the relationships between GM volume, WM volume and WM integrity (FA values) on the one hand, and fibrosis (score and area) and elevated levels of cytokines on the other hand. Results are reported at $p < 0.001$ (uncorrected for multiple comparisons) with a minimal cluster size (k) of 60 voxels (200 mm3).

Significant clusters of GM were labelled using the Harvard–Oxford cortical and subcortical structural atlases implemented in FSL (https://fsl.fmrib.ox.ac.uk/fsl/fslwiki/Atlases). WM regions and tracts were manually labelled using the MRI Atlas of Human White Matter (Oishi et al., 2011).

Preclinical data

Animal Subjects

The *in vivo* portion of the study with sP rats was performed at the Neuroscience Institute, Section of Cagliari, National Research Council of Italy. The experimental procedures employed in this study fully complied with European Directive no. 2010/63/EU and subsequent Italian

For Review Only

Legislative Decree no. 26, March 4, 2014, on the "Protection of animals used for scientific purposes".

We used male, 95th-generation sP rats that were approximately 75 days old at the start of the study. Rats were individually housed in standard plastic cages with wood chip bedding. Single-cage housing started 15 days before exposure to alcohol. The animal facility was under an inverted 12:12-hour light/dark cycle (lights on at 9:30 pm) at a constant temperature of 22 ± 2°C and a relative humidity of approximately 60%. Regular food pellets (Mucedola, Settimo Milanese, Italy) were always available. Alcohol was offered under the standard, home-cage 2-bottle choice regimen between an alcohol solution (10% in tap water, v/v) and tap water with unlimited access for 24 h/d. Under this procedure, sP rats usually consume daily approximately 6 g/kg pure alcohol with relatively steady intakes over time, modelling excessive alcohol drinking in humans (Colombo *et al.*, 2006). The left–right position of the 2 bottles was randomly interchanged to avoid the development of position preference. Alcohol and water intake were recorded once weekly. Alcohol-drinking rats (n = 6) were exposed to the "alcohol *vs* water" choice regimen for 50 consecutive weeks. Conversely, control rats (n = 6) were exposed to 2 bottles containing tap water.

At the end of the 50-week period of alcohol exposure, rats were killed by guillotine. Alcohol and water bottles were removed immediately before killing. Heads (after removal of the skin and muscles) and liver samples were immediately fixed in a solution of 2% paraformaldehyde and 0.2% picric acid phosphate buffer for 48h. Samples were then individually placed in 50mL falcon and rinsed with a solution of PBS with 20% sucrose followed by PBS with azide (0,01%) and stored at 4°C for further analysis.

Ex vivo brain MRI acquisitions

For Review Only

Imaging was carried out on a Pharmascan 7T/12 cm system using surface coils (Bruker, Germany). Anatomical images were acquired with high resolution sequence T2 RARE (Rapid acquisition with repetition enhancement) in 3D with the following parameters: TE 40 ms, TR 4500ms, resolution 0.1x0.1x0.1 mm3, thickness 0.1 mm, field of view 3.2x3.2x2.56, acquisition time 3h50. DTI data were acquired with the following parameters: TR 3500 ms, TE 23 ms, 32 diffusion directions,b=1048s/mm^2 64 horizontal slices were planned for every subject (field of view = 32x32 mm2, in-plane resolution = 0.2 x 0.2 mm2, slice thickness = 0.4mm).

MRI processing

Volumetric analyses were performed using ImageJ (http://imagej.nih.gov/ij), with manual delimitation of regions of interest (ROI) from one blinded experimenter (AL) and automatic calculation of these regional areas. To correctly delimit ROIs, we used the Paxinos-Watson rat brain atlas (Paxinos and Watson, 2013).

DTI analyses were conducted with the software DSI Studio (http://dsi-studio.labsolver.org). For each subject, the following parameter maps were computed: FA, mean diffusivity (MD), axial diffusivity (AD), radial diffusivity (RD). Whole brain tractography was calculated using a deterministic, tensor-based approach with the following parameters: FA>0.20 and angle threshold 45°.

Immunohistochemistry

Brain and liver portions were frozen in Tissue-Tek (Miles Scientifix, Naperville, IL, USA). Cryostat-cut sections (10µm) were collected on poly-lysine slides and stored at -80°C before processing.

Sections were co-incubated overnight with goat anti-rat Iba1 (1:1000, abcam ab5076), chicken anti-rat GFAP (1:2000, abcam ab4674), goat anti-rat collagen-IV (1:1000, Southern biotech 1340), rabbit anti-rat cleaved caspase 3 (1:1000, Cell signaling 9664-5), rabbit anti-rat

For Review Only

myeloperoxidase (1:200, abcam ab9535). Primary antibodies were revealed using Fab'2 fragments of donkey anti-goat linked to CY3 or FITC, anti-rabbit linked to CY3 or FITC, anti-chicken linked to CY5 (1:600 Jackson ImmunoResearch, West Grove, USA). Washed sections were coverslipped with antifade medium containing DAPI. Epifluorescence images were digitally captured using a LEICA DM6000 epifluorescence microscope-coupled coolsnap camera, visualized with Leica MM AF 2.2.0 software (Molecular devices, USA) and further processed using ImageJ. Specificity controls were performed by not adding primary antibodies.

Fluorojade C staining protocol was performed as previously described (Drieu *et al.*, 2020).

Statistical analysis

Preclinical data were analyzed using Mann-Whitney's tests to compare alcohol-drinking versus control (i.e., water-exposed) sP rats. P values <0.05 were considered statistically significant.

Data availability

All data and materials used within this study will be made available, upon reasonable

request, to research groups wishing to reproduce/confirm our results.

For Review Only

Results

Clinical data

Pattern of Brain Alterations in AUD patients

GM Volume

Compared to HC, AUD patients had significantly lower GM volume in frontal and prefrontal areas, insula, lateral, and medial temporal cortices, cingulate and occipital cortices, but also in subcortical regions including the thalamus, putamen, and caudate nuclei, and in the cerebellum ($p < 0.001$, uncorrected, $k = 60$). These results remained significant after correction for multiple comparisons but with smaller cluster sizes (family-wise error [FWE], $p < 0.05$; Fig. 1A).

WM Volume

Compared to HC, AUD patients had significantly lower WM volume in the entire corpus callosum (genu, body and splenium), corona radiata, cingulum, thalamus, and cerebellum ($p < 0.001$, uncorrected, $k = 60$). These results (except for the cerebellum) remained significant after correction for multiple comparisons but with smaller cluster sizes (family-wise error [FWE], $p < 0.05$; Fig. 1A).

WM integrity

Compared to HC, AUD patients had significantly lower FA values in a large set of fibers including the corpus callosum, the anterior corona radiata, the anterior limb of the internal capsule, the cingulum, the middle cerebellar peduncle, and the fornix ($p < 0.001$, uncorrected, $k = 60$). These results remained significant after correction for multiple comparisons but with smaller cluster size (FWE, $p < 0.05$; Fig. 1B).

Biological and inflammatory profiles of AUD

For Review Only

Compared to HC, AUD patients had liver dysfunctionwith elevated levels of GGT and higher ASAT/ALAT ratio (p=0.008; Fig. 1C). Fibrometer analysis revealed liver fibrosis in AUD patients with higher fibrosis score and area compared to HC (p=0.01 and p=0.0003respectively, Fig. 1D). There was no significant correlation between fibrosis (score or area) and alcohol history variables (AUDIT, alcohol misuse, daily alcohol consumption, Cushman score) in AUD patients (data not shown).

AUD patients also had elevated levels of TNF and IL-8 compared to HC (p=0.02 and p=0.009respectively; Fig. 1E).There was also no significant correlation between elevated circulating cytokines (IL-8 and TNF) and alcohol history variables (AUDIT, alcohol misuse, daily alcohol consumption, Cushman score) in AUD patients (data not shown).

Relationships between liver fibrosis and brain structure

Significant negative correlations (p <0.001, uncorrected, k = 60) were found between the fibrosis score and grey matter volume in the putamen (left and right) and cerebellum (lobules 4-5 and the vermis; Fig. 2A). A significant negative correlation (p <0.001, uncorrected, k = 60) was also found with the white matter volume in the angular gyrus and occipital cortex (Fig. 2B). There was no other significant correlation between liver fibrosis and brain structure.

Relationships between circulating cytokines, brain structure and liver function

Given the fact that only the levels of IL-8 and TNF were elevated in AUD compared with HC, we conducted correlations between brain structure and these cytokines only.

A significant negative correlation (p <0.001, uncorrected, k = 60) was found between IL-8 levels and grey matter volume in the orbitofrontal region (Fig. 2C). Significant positive correlations (p <0.001, uncorrected, k = 60) were found between TNF levels and grey matter volume in the prefrontal and temporal cortices and in the cerebellum (Fig 2C). There was no other significant correlation between these cytokines and brain structure.

For Review Only

We found a positive correlation between IL-8 levels on the one hand and fibrosis score (r=0,64; p=0.006) and fibrosis area (r=0,73; p<0,001; data not shown) on the other hand.

Preclinical data

Alcohol consumption

Over the 50-week period, weekly alcohol intake in alcohol-drinking rats averaged between 35 and 50 g/kg (Figure S2). These weekly intakes were suggestive of daily alcohol intakes of 5.0-7.5 g/kg, i.e. the standard daily amount of alcohol voluntarily consumed by male sP rats when exposed to the homecage 2-bottle "alcohol *vs* water" choice regimen with unlimited access (Colombo *et al.*, 2006). Weekly mean alcohol preference (defined as the ratio between the amounts of alcohol solution consumed and the total liquid intake) rose from 65% (Week 1) to 95% (from Week 7 onwards) (data not shown), reproducing a basal feature of alcohol drinking in sP rats (Colombo et al., 2006).

Impact of 50-week alcohol drinking on brain structure

The entire corpus callosum (genu, body, splenium) had significantly smaller volume in alcohol-drinking rats compared to control rats (p=0.031; Fig. 3C). There was no difference of volume in other brain regions (hippocampus, cerebellum, and total brain volume; Fig 3D-F).

Region-based tractography revealed microstructural alterations of the corpus callosum and fornix in alcohol-drinking rats compared to control rats. Fibers volume of the entire corpus callosum and more precisely in the body and splenium was significantly lower in alcohol-drinking rats compared to control rats (Fig 4B; F; G). FA in the genu of the corpus callosum was decreased in alcohol-drinking rats, although the difference did not reach statistical significance (p=0.06; Fig 4D). In the fornix, the volume of fibers was significantly lower in alcohol-drinking rats (p=0.004) and there was a trend for a higher MD (p=0.08; Fig 4J).

For Review Only

Impact of 50-week alcohol drinking on brain inflammation

We measured microglial (Iba1$^+$ staining, Fig. 5A) and astrocytic (GFAP$^+$ staining, Fig. 5D) density in the corpus callosum, cortex, and hippocampus by immunohistochemistry (IHC). The density of microglial cells (Iba1$^+$ cells) was significantly higher in alcohol-drinking rats in all these regions (Fig 5B). The number of GFAP$^+$ cells was also higher in alcohol-drinking rats. In the cortex and the hippocampus, the difference was statistically significant ($p<0.05$; Fig 5E); only a trend was found in the corpus callosum ($p<0.1$; Fig 5E).

Fluorojade C staining and caspase 3 did not reveal any neurodegeneration and apoptosis in alcohol-drinking and control rats (data not shown).

Impact of 50-week alcohol drinking on liver inflammation

We measured the number of Kupffer cells (KC) (resident macrophages in the liver) (Iba 1$^+$ cells; Fig 6A) and of hepatic stellate cells (GFAP$^+$ cells; Fig 6C). We found a higher number of KC in the liver of alcohol-drinking rats compared to control rats ($p<0.05$; Fig 6B).

The number of hepatic stellate cells (HSC) (main effectors of liver fibrosis) was similar in both groups (Fig 6E). However, the morphology of HSC was different in the two conditions: control rats showed ramified HSC cells (corresponding to a quiescent phenotype), whereas alcohol-drinking rats showed more ameboid HSC, suggestive of activated HSC (Fig 6D, 6F). When cells were ramified there was no difference in the number of ramifications per cell (data not shown).

Hematoxylin & Eosin staining revealed no major difference in the liver structure and indicated an absence of fibrosis, steatosis or neutrophil infiltration (Fig S1).

For Review Only

Discussion

The objective of the present study was to gain a better understanding of the contribution of inflammation and liver fibrosis to alcohol-related brain damage. The main novelty was to use a translational approach including a biological and neuroimaging investigation of AUD patients as well as an *ex-vivo* imaging and immunohistological examination of sP rats.

Brain and liver abnormalities, and systemic inflammation in AUD patients

In accordance with previous studies, we showed that AUD patients had macrostructural brain abnormalities in both GM and WM cortical and subcortical regions (Pitel *et al.*, 2012; Segobin *et al.*, 2019). We also found widespread microstructural alterations in AUD patients consistent with recent investigations (De Santis *et al.*, 2019; Segobin *et al.*, 2019). Even in the absence of cirrhosis, AUD patients had alcohol-related liver dysfunction (Mancinelli and Ceccanti, 2009; Ritz *et al.*, 2016).

For Review Only

Regarding inflammation, we did not find any difference in the circulating levels of the proinflammatory cytokines MCP-1 and MIP-1b between controls and AUD patients, in contrast to previous studies (Afford *et al.*, 1998; Fisher *et al.*, 1999; Leach *et al.*, 2016; Grzegorzewska *et al.*, 2017). It is worthwhile mentioning that these previous studies were conducted in patients with alcoholic advanced liver disease, hepatitis or non-alcoholic steatohepatitis, whereas patients included in the present study did not exhibit any ostensible clinical signs of liver disease. Despite the fact that previous studies have found that MCP-1 could be a biomarker of liver disease, our data revealed that this chemokine is not related to early or discrete liver dysfunction.

In accordance with previous studies (Laso *et al.*, 2007; Leclercq *et al.*, 2014), we found higher plasmatic levels of the pro-inflammatory cytokines IL-8 and TNF in AUD patients compared to HC. Serum TNF concentration was reported to be higher in AUD patients than in

the general population, regardless of the level of alcohol consumption (Gonzalez-Quintela *et al.*, 2008; Heberlein *et al.*, 2014) and to decrease within the first days of abstinence (Umhau *et al.*, 2014; Girard *et al.*, 2019). By contrast, IL-8, which is not primarily associated with acute inflammation and rather acts as a potential angiogenic factor and chemotactic attractor for all known types of migratory immune cells, remains increased at least for the first 6 months after alcohol withdrawal (Girard *et al.*, 2019).

Liver impairment and systemic inflammation are associated with regional brain volume

While systemic inflammation is known to be associated with cognitive impairments and cerebral damage in cirrhotic patients in the case of encephalic encephalopathy, studies of these relationships in non-cirrhotic patients are scarce. Some findings suggested that systemic inflammation and liver fibrosis correlate with behavioral impairments in non-cirrhotic patients (Leclercq *et al.*, 2012, 2014; Ritz *et al.*, 2016) but, to our knowledge, there is no study on the potential relationship between systemic inflammation and structural brain damage.

The present results showed that circulating proinflammatory cytokines and liver fibrosis correlated with brain macrostructurein recently detoxified AUD patients without ostensible liver damage. We found a specific vulnerability of putamen toliver fibrosis. Accordingly, the basal ganglia volume, including the putamen, is altered in severe liver disease (Lin *et al.*, 2012). Liver function has also been related to brain atrophy (Chen *et al.*, 2012). However, a major limitation of that previous study was that liver function was only assessed with GGT and transaminases levels, which are not specific to liver fibrosis or alcohol-related liver damage. In addition to a clear relationship between liver function and specific regional brain volume, we show here that levels of circulating proinflammatory cytokines were associated with brain volumes, as previously described in schizophrenia (Wu *et al.*, 2019). Proinflammatory

For Review Only

cytokines could come from the liver (Gao *et al.*, 2019) and in particular from activated KC. One possible mechanistic hypothesis is that alcohol could directly (or indirectly) activate KC, which would initiate the synthesis of proinflammatory cytokines that would be released to the bloodstream and reach the brain.

In a previous study, chronic alcohol drinking induced increased levels of LPS in the serum of sP rats and altered their intestinal microbiota composition (Posteraro *et al.*, 2018). These results have also been shown in alcohol non-preferring rats and AUD patients (Leclercq *et al.*, 2017). Several studies have shown that increased LPS circulating levels induced by alcohol stimulate KC to generate ROS and cytokines (Lamas-Paz *et al.*, 2018). These inflammatory mediators subsequently activate HSCs via a Toll-like receptor 4 (TLR4) signaling pathway, which eventually results in enhanced, chronic production of ECM proteins - and promotion of fibrogenesis (Seki *et al.*, 2007; Jagavelu *et al.*, 2010). Additionally, HSCs are also enriched with TLR4 to which LPS directly binds, and can thus activate through LPS signaling (Inokuchi *et al.*, 2011).

Chronic alcohol exposure induced similar brain abnormalities in a rat model of AUD

sP rats have been selectively bred for high alcohol preference and consumption. Notably, sP rats meet all the fundamental requirements posed when defining an animal model of AUD, including tolerance and behavioral dependence (Colombo *et al.*, 2006; Bell *et al.*, 2012). The results collected in sP rats were similar to those observed in AUD patients in terms of macrostructural and microstructural brain alterations, in line with previous preclinical investigations (Pfefferbaum *et al.*, 2006*a*; De Santis *et al.*, 2019). Chronic (50 consecutive weeks) alcohol drinking resulted in a lower WM volume in the fornix and corpus callosum,

For Review Only

potentially reflecting myelin and/or axonal damage or glial/cellular reaction during neuroinflammation phenomenon (De Santis *et al.*, 2019).

Chronic alcohol drinking induces brain and liver inflammatory responses

We describe here, for the first time, an alcohol-induced inflammatory response in the brain of sP rats after 50 consecutive weeks of voluntary alcohol drinking. Importantly, this inflammatory response was not accompanied by signs of neurodegeneration or apoptosis. Chronic alcohol drinking induces innate immune signaling cascades through the activation of the proinflammatory transcription factor,NF-κB (Crews *et al.*, 2006, 2011; Vallés *et al.*, 2006). We found a significant higher number in microglia and astrocytes in all the brain regions studied (cortex, corpus callosum, and hippocampus). In addition, microglial cells in alcohol-drinking rats showed morphological alterations characterized by a transition from a resting and ramified morphology, to an amoeboid phenotype compatible with a partially activated state. Several studies have proposed that partially activated microglia is necessary in neuroprotection and axonal regeneration (Wainwright *et al.*, 2009; Shokouhi *et al.*, 2010), an assumption compatible with the absence of neurodegeneration observed in the present study.

Fifty weeks of alcohol drinking provoked a series of inflammatory responses in the liver of sP rats, including KC proliferation and HSC activation. These responses were not accompanied by clear signs of liver damage (no significant steatosis, fibrosis, or neutrophil infiltration). These results indicate that chronic alcohol consumption induces inflammatory responses in the liver even in absence of hepatitis or cirrhosis, with activation of HSC. These cells, in addition of playing a role in the development of steatosis and fibrosis, are major producers of cytokines and chemokines that are released to the bloodstream. Chronic alcohol consumption may thus directly provoke inflammatory responses in the brain and indirectly through the trigger of peripheral cytokines reaching the brain. In agreement with this

For Review Only

hypothesis, we found a correlation between peripheral cytokine levels and brain volumes in AUD patients as well as microglial activation in a rodent model of AUD.

Limitations and future perspectives

One major limitation of this study is the absence of information about cytokines levels in the serum or in the brain of sP rats. A recent study using sP rats of the same cohort used in the present study found similar levels of TNF in the serum of control and alcohol-drinking rats (Posteraro *et al.*, 2018), which would suggest an independent effect of alcohol on the brain and liver in terms of inflammatory responses.

Also, other factors, such as genetics, dietary status, gut permeability, and the intestinal biome, could play a role on liver inflammation and the development of neuroinflammation and neurodegeneration (Kirpich *et al.*, 2008; Leclercq *et al.*, 2012). It has been shown that, in sP rats, the expression of neuropeptides and microglia activation were altered in several brain regions compared to the alcohol-nonpreferring counterpart, indicating preexisting genetic differences in alcohol-induced responses (Rossetti *et al.*, 2019).

Our results show that alcohol provokes liver and brain inflammatory responses that correlate with brain abnormalities even in the absence of advanced liver damage in both AUD patients and alcohol-preferring sP rats consuming alcohol chronically. Future studies are needed to elucidate causality links regarding the brain-liver interactions (i.e., whether alcohol-induced liver and brain inflammatory responses arise independently or whether liver and brain inflammatory responses are related), in order to find new therapeutic strategies aiming at blocking alcohol-related brain damage.

For Review Only

References:

Afford SC, Fisher NC, Neil DAH, Fear J, Brun P, Hubscher SG, et al. Distinct patterns of chemokine expression are associated with leukocyte recruitment in alcoholic hepatitis and alcoholic cirrhosis. J Pathol 1998; 186: 82–9.

Alfonso-Loeches S, Ureña-Peralta J, Morillo-Bargues MJ, Gómez-Pinedo U, Guerri C. Ethanol-induced TLR4/NLRP3 neuroinflammatory response in microglial cells promotes leukocyte infiltration across the BBB. Neurochem Res 2016; 41: 193–209.

American Psychiatric Association. Diagnostic criteria from DSM-IV. The Association; 1994

American Psychiatric Association. American Psychiatric Association explains DSM-5. BMJ 2013; 346

American Psychological Association (APA). Diagnostic and Statistical Manual of Mental Disorders: Depressive Disorders [Internet]. American Psychiatric Publishing, Inc; 2013[cited 2020 May 7] Available from: http://dsm.psychiatryonline.org//content.aspx?bookid=556&sectionid=41101760

Beck AT, Ward CH, Mendelson M, Mock J, Erbaugh J. An Inventory for Measuring Depression. Arch Gen Psychiatry 1961; 4: 561–71.

Bell RL, Sable HJK, Colombo G, Hyytia P, Rodd ZA, Lumeng L. Animal models for medications development targeting alcohol abuse using selectively bred rat lines: Neurobiological and pharmacological validity. Pharmacol Biochem Behav 2012; 103: 119–55.

Calès P, Oberti F, Michalak S, Hubert-Fouchard I, Rousselet MC, Konaté A, et al. A novel panel of blood markers to assess the degree of liver fibrosis. Hepatology 2005; 42: 1373–81.

Chanraud S, Reynaud M, Wessa M, Penttilä J, Kostogianni N, Cachia A, et al. Diffusion

For Review Only

tensor tractography in mesencephalic bundles: Relation to mental flexibility in detoxified alcohol-dependent subjects. Neuropsychopharmacology 2009; 34: 1223–32.

Chen CH, Walker J, Momenan R, Rawlings R, Heilig M, Hommer DW. Relationship Between Liver Function and Brain Shrinkage in Patients with Alcohol Dependence. Alcohol Clin Exp Res 2012; 36: 625–32.

Colombo G, Lobina C, Carai MAM, Gessa GL. Phenotypic characterization of genetically selected Sardinian alcohol-preferring (sP) and -non-preferring (sNP) rats. Addict Biol 2006; 11: 324–38.

Crews FT, Bechara R, Brown LA, Guidot DM, Mandrekar P, Oak S, et al. Cytokines and Alcohol. Alcohol Clin Exp Res 2006; 30: 720–30.

Crews FT, Zou J, Qin L. Induction of innate immune genes in brain create the neurobiology of addiction. Brain Behav Immun 2011; 25

Cushman P, Lerner W, Cramer M. Adrenergic agonist therapy in alcohol withdrawal states in man. Psychopharmacol Bull 1985; 21: 651–6.

D'Mello C, Swain MG. Immune-to-brain communication pathways in inflammation-associated sickness and depression. In: Current Topics in Behavioral Neurosciences. Springer Verlag; 2017. p. 73–94

Drieu A, Lanquetin A, Levard D, Glavan M, Campos F, Quenault A, et al. Alcohol exposure–induced neurovascular inflammatory priming impacts ischemic stroke and is linked with brain perivascular macrophages. JCI Insight 2020; 5

Fisher NC, Neil DAH, Williams A, Adams DH. Serum concentrations and peripheral secretion of the beta chemokines monocyte chemoattractant protein 1 and macrophage inflammatory protein 1 α in alcoholic liver disease. Gut 1999; 45: 416–20.

For Review Only

Frischknecht U, Hermann D, Tunc-Skarka N, Wang GY, Sack M, van Eijk J, et al. Negative Association Between MR-Spectroscopic Glutamate Markers and Gray Matter Volume After Alcohol Withdrawal in the Hippocampus: A Translational Study in Humans and Rats. Alcohol Clin Exp Res 2017; 41: 323–33.

Gache P, Michaud P, Landry U, Accietto C, Arfaoui S, Wenger O, et al. The alcohol use disorders identification test (AUDIT) as a screening tool for excessive drinking in primary care: Reliability and validity of a french version. Alcohol Clin Exp Res 2005; 29: 2001–7.

Gao B, Ahmad MF, Nagy LE, Tsukamoto H. Inflammatory pathways in alcoholic steatohepatitis. J Hepatol 2019; 70: 249–59.

Girard M, Malauzat D, Nubukpo P. Serum inflammatory molecules and markers of neuronal damage in alcohol-dependent subjects after withdrawal. World J Biol Psychiatry 2019; 20: 76–90.

Gonzalez-Quintela A, Alende R, Gude F, Campos J, Rey J, Meijide LM, et al. Serum levels of immunoglobulins (IgG, IgA, IgM) in a general adult population and their relationship with alcohol consumption, smoking and common metabolic abnormalities. Clin Exp Immunol 2008; 151: 42–50.

Grzegorzewska AE, Świderska MK, Mostowska A, Warchoł W, Jagodziński PP. Polymorphisms of T helper cell cytokine-associated genes and survival of hemodialysis patients - a prospective study. BMC Nephrol 2017; 18

Halvorson MR, Campbell JL, Sprague G, Slater K, Noffsinger JK, Peterson CM. Comparative Evaluation of the Clinical Utility of Three Markers of Ethanol Intake: The Effect of Gender. Alcohol Clin Exp Res 1993; 17: 225–9.

Harper CG, Kril JJ. Corpus callosal thickness in alcoholics. Br J Addict 1988; 83: 577–80.

For Review Only

Heberlein A, Schuster R, Ziert Y, Opfermann B, Bleich S, Hillemacher T. The implications for the biological and sociodynamic causal explanations of attitudes toward alcohol-dependent patients. Psychiatry Res 2014; 215: 766–70.

Inokuchi S, Tsukamoto H, Park E, Liu ZX, Brenner DA, Seki E. Toll-like receptor 4 mediates alcohol-induced steatohepatitis through bone marrow-derived and endogenous liver cells in mice. Alcohol Clin Exp Res 2011; 35: 1509–18.

Jagavelu K, Routray C, Shergill U, O'Hara SP, Faubion W, Shah VH. Endothelial cell toll-like receptor 4 regulates fibrosis-associated angiogenesis in the liver. Hepatology 2010; 52: 590–601.

Junghanns K. MILDLY DISTURBED HEPATIC AND PANCREATIC FUNCTION DURING EARLY ABSTENTION FROM ALCOHOL IS ASSOCIATED WITH BRAIN ATROPHY AND WITH DISTURBED PSYCHOMETRIC PERFORMANCE. Alcohol Alcohol 2004; 39: 113–8.

Jurica, P. J., Leitten, C. L., & Mattis S. Dementia Rating Scale—2 professional manual. Psychol Assess Resour 2001

Kalk NJ, Guo Q, Owen D, Cherian R, Erritzoe D, Gilmour A, et al. Decreased hippocampal translocator protein (18kDa) expression in alcohol dependence: A [11C]PBR28 PET study. Transl Psychiatry 2017; 7

Kim SW, Wiers CE, Tyler R, Shokri-Kojori E, Jang YJ, Zehra A, et al. Influence of alcoholism and cholesterol on TSPO binding in brain: PET [11 C]PBR28 studies in humans and rodents. Neuropsychopharmacology 2018; 43: 1832–9.

Kirpich IA, Solovieva N V., Leikhter SN, Shidakova NA, Lebedeva O V., Sidorov PI, et al. Probiotics restore bowel flora and improve liver enzymes in human alcohol-induced liver

For Review Only

injury: a pilot study. Alcohol 2008; 42: 675–82.

Koike H, Mori K, Misu K, Hattori N, Ito H, Hirayama M, et al. Painful alcoholic polyneuropathy with predominant small-fiber loss and normal thiamine status. Neurology 2001; 56: 1727–32.

Lamas-Paz A, Hao F, Nelson LJ, Vázquez MT, Canals S, del Moral MG, et al. Alcoholic liver disease: Utility of animal models. World J Gastroenterol 2018; 24: 5063–75.

Laso FJ, Vaquero JM, Almeida J, Marcos M, Orfao A. Production of inflammatory cytokines by peripheral blood monocytes in chronic alcoholism: Relationship with ethanol intake and liver disease. Cytom Part B - Clin Cytom 2007; 72: 408–15.

Leach N V., Dronca E, Craciun EC, Crisan D. High levels of serum homocysteine in non-alcoholic steatohepatitis. Eur J Intern Med 2016; 35: e38–9.

Leclercq S, Cani PD, Neyrinck AM, Stärkel P, Jamar F, Mikolajczak M, et al. Role of intestinal permeability and inflammation in the biological and behavioral control of alcohol-dependent subjects. Brain Behav Immun 2012; 26: 911–8.

Leclercq S, Matamoros S, Cani PD, Neyrinck AM, Jamar F, Stärkel P, et al. Intestinal permeability, gut-bacterial dysbiosis, and behavioral markers of alcohol-dependence severity. Proc Natl Acad Sci U S A 2014; 111: E4485–93.

Leclercq S, De Timary P, Delzenne NM, Stärkel P. The link between inflammation, bugs, the intestine and the brain in alcohol dependence. Transl Psychiatry 2017; 7

Lin WC, Chou KH, Chen CL, Chen CH, Chen HL, Feekes JA, et al. Significant volume reduction and shape abnormalities of the basal ganglia in cases of chronic liver cirrhosis. Am J Neuroradiol 2012; 33: 239–45.

Mackey S, Allgaier N, Chaarani B, Spechler P, Orr C, Bunn J, et al. Mega-analysis of gray

For Review Only

matter volume in substance dependence: General and substance-specific regional effects. Am J Psychiatry 2019; 176: 119–28.

Mancinelli R, Ceccanti M. Biomarkers in Alcohol Misuse: Their Role in the Prevention and Detection of Thiamine Deficiency. Alcohol Alcohol 2009; 44: 177–82.

Montesinos J, Alfonso-Loeches S, Guerri C. Impact of the Innate Immune Response in the Actions of Ethanol on the Central Nervous System. Alcohol Clin Exp Res 2016; 40: 2260–70.

Paxinos G, Watson C. The Rat Brain in Stereotaxic Coordinates : Hard Cover Edition. Elsevier Science; 2013

Pfefferbaum A, Adalsteinsson E, Sood R, Mayer D, Bell R, McBride W, et al. Longitudinal brain magnetic resonance imaging study of the alcohol-preferring rat. Part II: Effects of voluntary chronic alcohol consumption. Alcohol Clin Exp Res 2006; 30: 1248–61.

Pfefferbaum A, Adalsteinsson E, Sullivan E V. Dysmorphology and microstructural degradation of the corpus callosum: Interaction of age and alcoholism. Neurobiol Aging 2006; 27: 994–1009.

Pfefferbaum A, Lim KO, Desmond JE, Sullivan E V. Thinning of the Corpus Callosum in Older Alcoholic Men: A Magnetic Resonance Imaging Study. Alcohol Clin Exp Res 1996; 20: 752–7.

Pfefferbaum A, Rosenbloom M, Rohlfing T, Sullivan E V. Degradation of Association and Projection White Matter Systems in Alcoholism Detected with Quantitative Fiber Tracking. Biol Psychiatry 2009; 65: 680–90.

Pfefferbaum A, Sullivan E V. Microstructural but not macrostructural disruption of white matter in women with chronic alcoholism. Neuroimage 2002; 15: 708–18.

Pfefferbaum A, Sullivan E V. Disruption of brain white matter microstructure by excessive

For Review Only

intracellular and extracellular fluid in alcoholism: Evidence from diffusion tensor imaging. Neuropsychopharmacology 2005; 30: 423–32.

Pfefferbaum A, Sullivan E V., Hedehus M, Adalsteinsson E, Lim KO, Moseley M. In vivo detection and functional correlates of white matter microstructural disruption in chronic alcoholism. Alcohol Clin Exp Res 2000; 24: 1214–21.

Pitel AL, Chanraud S, Sullivan E V., Pfefferbaum A. Callosal microstructural abnormalities in Alzheimer's disease and alcoholism: Same phenotype, different mechanisms. Psychiatry Res - Neuroimaging 2010; 184: 49–56.

Pitel AL, Chételat G, Le Berre AP, Desgranges B, Eustache F, Beaunieux H. Macrostructural abnormalities in Korsakoff syndrome compared with uncomplicated alcoholism. Neurology 2012; 78: 1330–3.

Pitel AL, Segobin SH, Ritz L, Eustache F, Beaunieux H. Thalamic abnormalities are a cardinal feature of alcohol-related brain dysfunction. Neurosci Biobehav Rev 2015; 54: 38–45.

Posteraro B, Paroni Sterbini F, Petito V, Rocca S, Cubeddu T, Graziani C, et al. Liver Injury, Endotoxemia, and Their Relationship to Intestinal Microbiota Composition in Alcohol-Preferring Rats. Alcohol Clin Exp Res 2018; 42: 2313–25.

Qin L, He J, Hanes RN, Pluzarev O, Hong JS, Crews FT. Increased systemic and brain cytokine production and neuroinflammation by endotoxin following ethanol treatment. J Neuroinflammation 2008; 5: 10.

Rehm J, Allamani A, Aubin H-J, Della Vedova R, Elekes Z, Frick U, et al. People with Alcohol Use Disorders in Specialized Care in Eight Different European Countries. Alcohol Alcohol 2015; 50: 310–8.

For Review Only

Ritz L, Coulbault L, Lannuzel C, Boudehent C, Segobin S, Eustache F, et al. Clinical and Biological Risk Factors for Neuropsychological Impairment in Alcohol Use Disorder. PLoS One 2016; 11

Rossetti I, Zambusi L, Maccioni P, Sau R, Provini L, Paola Castelli M, et al. Predisposition to alcohol drinking and alcohol consumption alter expression of calcitonin gene-related peptide, neuropeptide Y, and microglia in bed nucleus of stria terminalis in a subnucleus-specific manner. Front Cell Neurosci 2019; 13

De Santis S, Bach P, Pérez-Cervera L, Cosa-Linan A, Weil G, Vollstädt-Klein S, et al. Microstructural White Matter Alterations in Men with Alcohol Use Disorder and Rats with Excessive Alcohol Consumption during Early Abstinence. JAMA Psychiatry 2019; 76: 749–58.

Segobin S, Laniepce A, Ritz L, Lannuzel C, Boudehent C, Cabé N, et al. Dissociating thalamic alterations in alcohol use disorder defines specificity of Korsakoff's syndrome. Brain 2019; 142: 1458–70.

Segobin S, Ritz L, Lannuzel C, Boudehent C, Vabret F, Eustache F, et al. Integrity of white matter microstructure in alcoholics with and without Korsakoff's syndrome. Hum Brain Mapp 2015; 36: 2795–808.

Seki E, De Minicis S, Österreicher CH, Kluwe J, Osawa Y, Brenner DA, et al. TLR4 enhances TGF-β signaling and hepatic fibrosis. Nat Med 2007; 13: 1324–32.

Shokouhi BN, Wong BZY, Siddiqui S, Lieberman AR, Campbell G, Tohyama K, et al. Microglial responses around intrinsic CNS neurons are correlated with axonal regeneration. BMC Neurosci 2010; 11

Tyler RE, Kim SW, Guo M, Jang YJ, Damadzic R, Stodden T, et al. Detecting

For Review Only

neuroinflammation in the brain following chronic alcohol exposure in rats: A comparison between in vivo and in vitro TSPO radioligand binding. Eur J Neurosci 2019; 50: 1831–42.

Umhau JC, Schwandt M, Solomon MG, Yuan P, Nugent A, Zarate CA, et al. Cerebrospinal fluid monocyte chemoattractant protein-1 in alcoholics: Support for a neuroinflammatory model of chronic alcoholism. Alcohol Clin Exp Res 2014; 38: 1301–6.

Vallés SL, Blanco AM, Pascual M, Guerri C. Chronic Ethanol Treatment Enhances Inflammatory Mediators and Cell Death in the Brain and in Astrocytes. Brain Pathol 2006; 14: 365–71.

Wainwright DA, Xin J, Mesnard NA, Beahrs TR, Politis CM, Sanders VM, et al. Exacerbation of Facial Motoneuron Loss after Facial Nerve Axotomy in CCR3-Deficient Mice. ASN Neuro 2009; 1: AN20090017.

Wilson J, Tay RY, McCormack C, Allsop S, Najman J, Burns L, et al. Alcohol consumption by breastfeeding mothers: Frequency, correlates and infant outcomes. Drug Alcohol Rev 2017; 36: 667–76.

Wu D, Lv P, Li F, Zhang W, Fu G, Dai J, et al. Association of peripheral cytokine levels with cerebral structural abnormalities in schizophrenia. Brain Res 2019; 1724

Zhao YN, Wang F, Fan YX, Ping GF, Yang JY, Wu CF. Activated microglia are implicated in cognitive deficits, neuronal death, and successful recovery following intermittent ethanol exposure. Behav Brain Res 2013; 236: 270–82.

For Review Only

Table legends:

Table 1 : Demographical and clinical description of the Healthy Controls (HC) and Alcohol Use Disorder patients (AUD)

Figure legends

Figure 1: Brain alterations, hepatic dysfunction, liver fibrosis and peripheral inflammation in HC and in AUD patients.

(A) AUD patients have significant gray matter (GM), and white matter (WM), shrinkage, as well as altered WM integrity (B) compared to healthy controls (HC). Larger images indicate a p-value of $p < 0.001$ uncorrected, and smaller images display the results using a restrictive $p < 0.05$ corrected for FWE to highlight the most significant regions (C) AUD patients had hepatic dysfunction with elevated levels of serum GGT and ASAT/ALAT ratio compared to HC. (D) Fibrometer® parameters showed liver fibrosis only in AUD patients. (E) AUD patients had significant elevated serum cytokine (TNF and IL8) compared to HC.

* $p < 0.05$, ** $p < 0.01$, *** $p < 0.001$ Mann-Whitney's test (or t-test only for log GGT)

Figure 2: Correlations between brain alterations, liver fibrosis and inflammation in AUD patients

In AUD, negative correlations between the fibrosis score and GM volume (A), WM volume (B) at $p < 0.001$ uncorrected. In AUD, negative correlations between cytokines (negative for IL-8 in purple and positive for TNF in blue) and GM volume (C) and an absence of correlation with WM volume (D) at $p < 0.001$. Cluster size: >60 voxels.

Figure 3: Chronic alcohol consumption specifically reduces the volume of the corpus callosum in sP rats.

For Review Only

(A) Representative scan of brain MRI (coronal view) with total brain, corpus callosum and hippocampus (delimited respectively in blue, red, and yellow). (B) Representative scan of brain MRI (sagittal view) used to measure cerebellum volume (delimited in pink). Total volume of the corpus callosum (C), hippocampus (D), total brain (E) and cerebellum (F) in control and alcohol-drinking rats. * $p < 0.05$, Mann-Whitney's test.

Figure 4: Chronic alcohol drinking impairs both corpus callosum volume and fiber integrity in sP rats.

(A) Individual reconstruction of fiber tracts in the entire corpus callosum. (B) White matter (WM) microstructure parameters in the entire corpus callosum of control and alcohol-drinking sP rats.

(C) Individual reconstruction of fiber tracts in the genu of the corpus callosum. (D) WM microstructure parameters in the genu of the corpus callosum of control and alcohol- drinking sP rats.

(E) Individual reconstruction of fiber tracts in the body of the corpus callosum. (F) WM microstructure parameters in the body of the corpus callosum of control and alcohol- drinking sP rats.

(G) Individual reconstruction of fiber tracts in the splenium of the corpus callosum. (H) WM microstructure parameters in the splenium of the corpus callosum of control and alcohol-drinking sP rats.

(I) Individual reconstruction of fiber tracts in the entire fornix (left and right). (J) WM microstructure parameters in the entire fornix of control and alcohol- drinking sP rats.

* $p < 0.05$, ** $p<0.01$, Mann-Whitney test.

For Review Only

Figure 5: Neuroinflammatory responses to chronic alcohol drinking in sP rats: microgliosis and astrogliosis in the corpus callosum, brain cortex and hippocampus, in the absence of neuronal death.

(A) Representative photomicrographs of microglia (Iba1+ cells) in the corpus callosum (delimited with dotted line), cortex and hippocampus in control and alcohol- drinking sP rats. Scale bar 50µm. (B) Quantification of microglial density. (C) Representative photomicrographs of microglial cell morphology in control and alcohol- drinking sP rats. Note the decreased process density (arrows) in alcohol-drinking rats. Scale bar 10µm. (D) Representative photomicrographs of astrocytes (GFAP+ cells) in the corpus callosum (delimited with dotted line), cortex and hippocampus of control and alcohol-drinking sP rats. Scale bar 50µm. (E) Quantification of astrocytic density. Scale bar 100µm # $p < 0.1$, * $p < 0.05$, ** $p<0.01$, Mann-Whitney test.

Figure 6: Chronic alcohol drinking **provokes liver inflammation in sP rats.**

(A) Representative photomicrographs of Kuppfer cells (Iba1+) in the liver of control and alcohol-drinking sP rats. Scale bar 100µm. (B) Quantification of Kuppfer cell density. (C) Representative photomicrograph of hepatic stellate cells (HSC) (GFAP+) in the liver of control and alcohol-drinking sP rats. Scale bar 100µm. (D) Detail of HSC. Note the ameboid shape in the alcohol-drinking condition, characteristic of the activated state of HSC. Scale bar 10µm. (E) Quantification of HSC density. (F) Percentage of ameboid HSC in the liver of alcohol-drinking and control sP rats. * $p < 0.05$, Mann-Whitney test.

Supplementary figure 1: Hematoxylin & Eosin staining in liver of control and alcohol-drinking sP rats.

(A) Representative photomicrographs of liver after hematoxylin & eosin staining. Scale bar 100µm.

For Review Only

Supplementary figure 2: Weekly alcohol drinking pattern in sP rats.

Weekly alcohol intake in sP rats exposed to the standard, homecage 2-bottle "alcohol (10% v/v) *vs* water" choice regimen with unlimited access (24 hours/day) for 50 consecutive weeks. Weekly alcohol intake is expressed in g/kg pure alcohol. Each point is the mean ± SEM of *n*=6 rats. $F(49,245)=6.53$, $P<0.005$ (1-way ANOVA with repeated measures).

For Review Only

		AUD N=25	HC N=16	P value
Demography	Age (years)	46.84 (8.586)	46.38 (6.531)	0.8543
	Men/Women Ratio	20/5	13/3	0.8718*
	Education (years of schooling)	12.04 (1.881)	11.69 (2.33)	0.5816
Clinical variables	DRS (total score /144)	135.4 (8.675)	141.4 (2.159)	**0.0021**
	BDI	10.68 (7.454)	3.625 (3.008)	**0.0006**
	STAI-A	29.74 (10.69)[b]	27.50 (7.220)	0.6555
	STAI-B	43.39 (12.07)[b]	33.88 (6.365)	**0.0174**
	AUDIT	29.08 (6.788)	2.875 (1.668)	**<0.0001**
	Alcohol misuse (number of years)	19.667 (8.91)	NA	/
	Daily alcohol consumption (units)	19.864 (8.91)	NA	/
	Length of sobriety (days)	11.42 (4.94)	NA	/

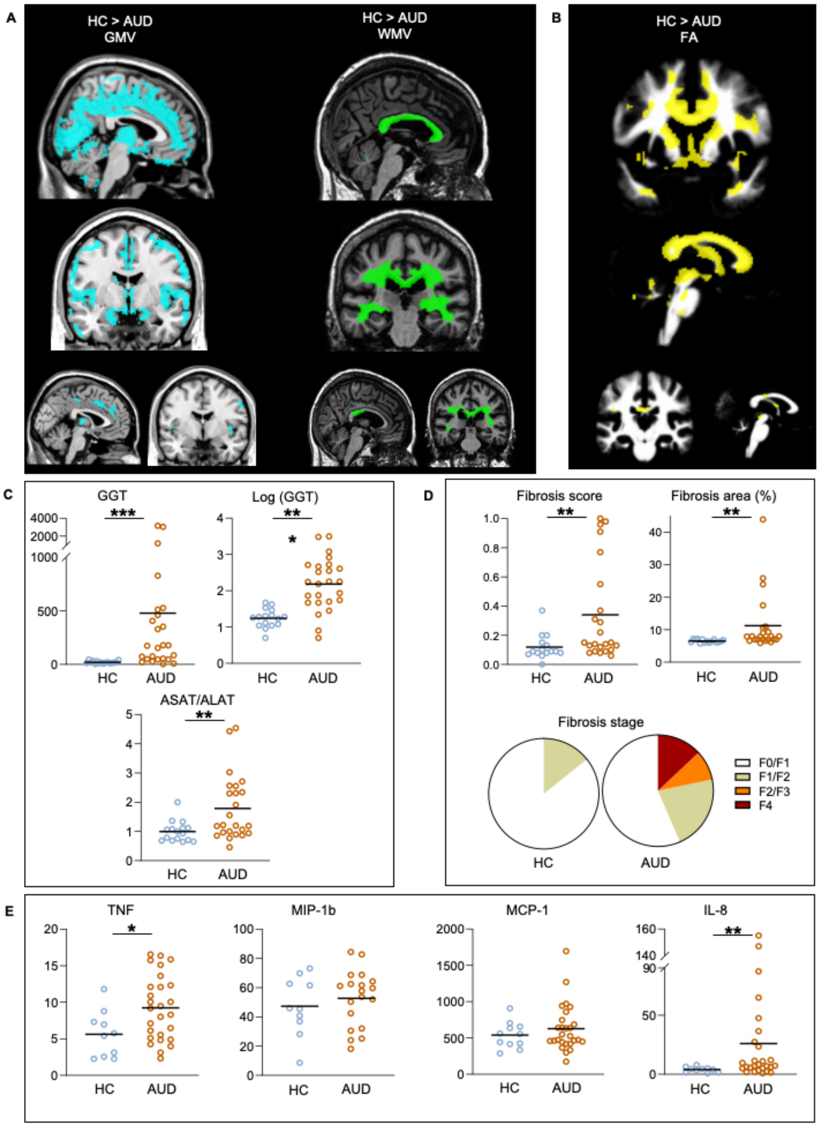

Figure 1- Brain alterations, hepatic dysfunction, liver fibrosis and peripheral inflammation in HC and in AUD patients.

190x275mm (96 x 96 DPI)

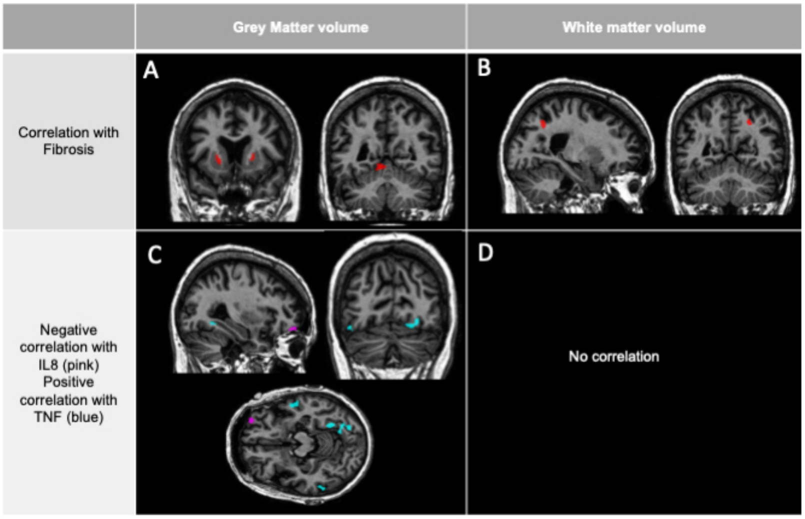

Figure 2- Correlations between brain alterations, liver fibrosis and inflammation in AUD patients

210x290mm (87 x 87 DPI)

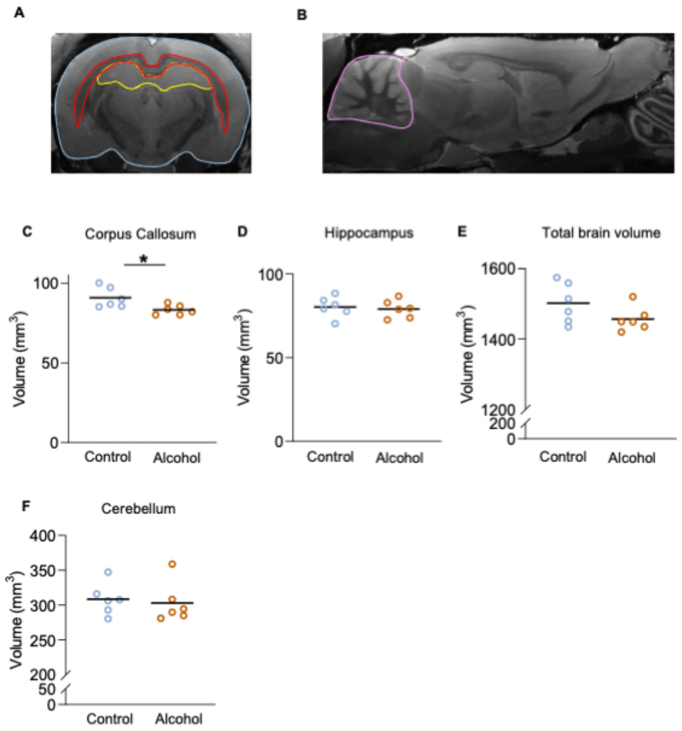

Figure 3- Chronic alcohol consumption specifically reduces the volume of the corpus callosum in sP rats

210x290mm (87 x 87 DPI)

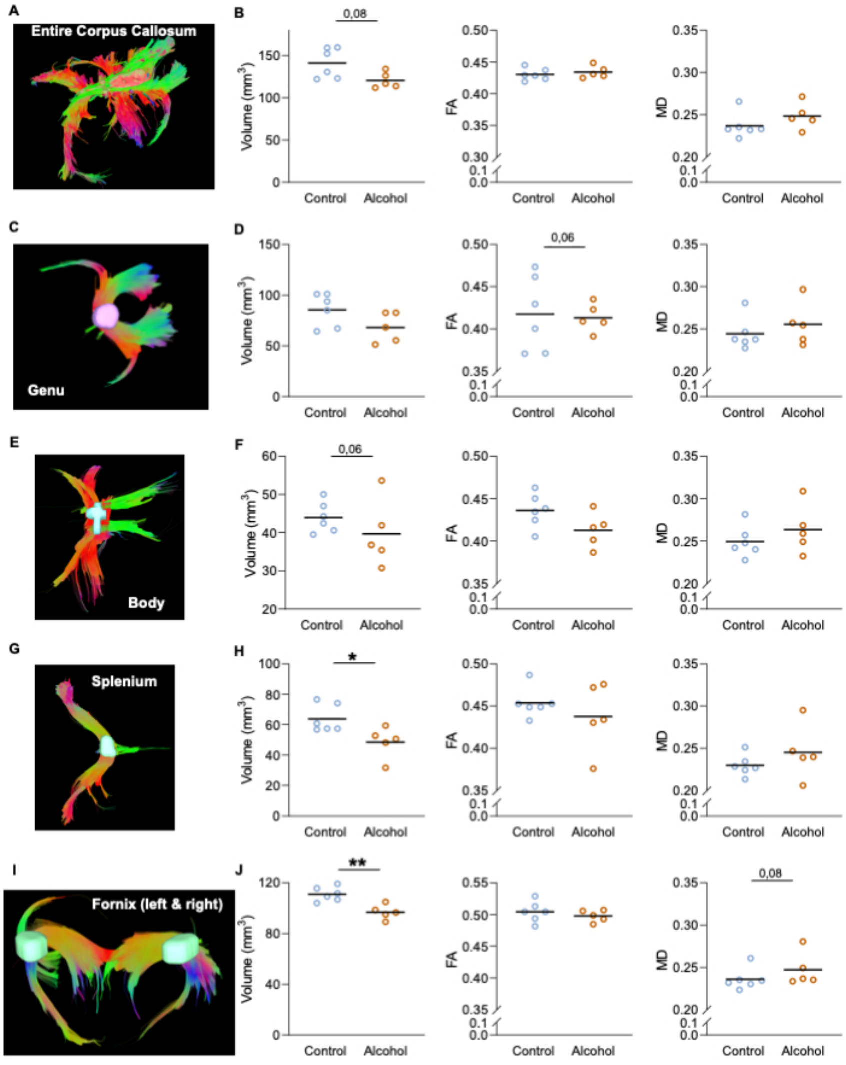

Figure 4- Chronic alcohol drinking impairs both corpus callosum volume and fiber integrity in sP rats.

210x290mm (87 x 87 DPI)

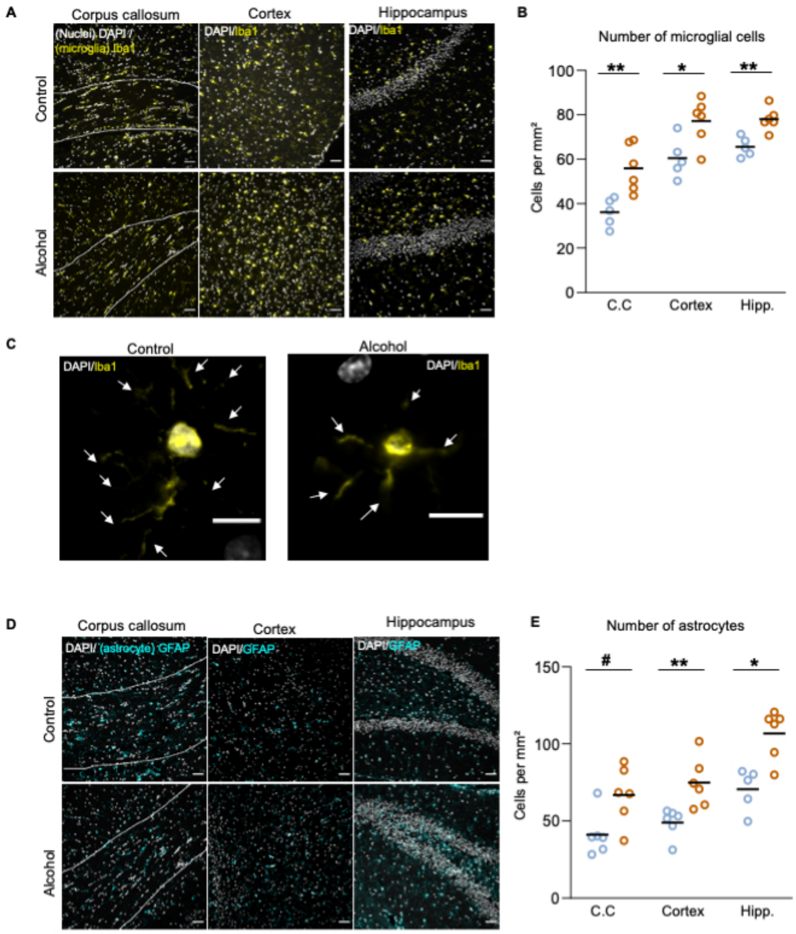

Figure 5- Neuroinflammatory responses to chronic alcohol drinking in sP rats: microgliosis and astrogliosis in the corpus callosum, brain cortex and hippocampus, in the absence of neuronal death.

210x290mm (87 x 87 DPI)

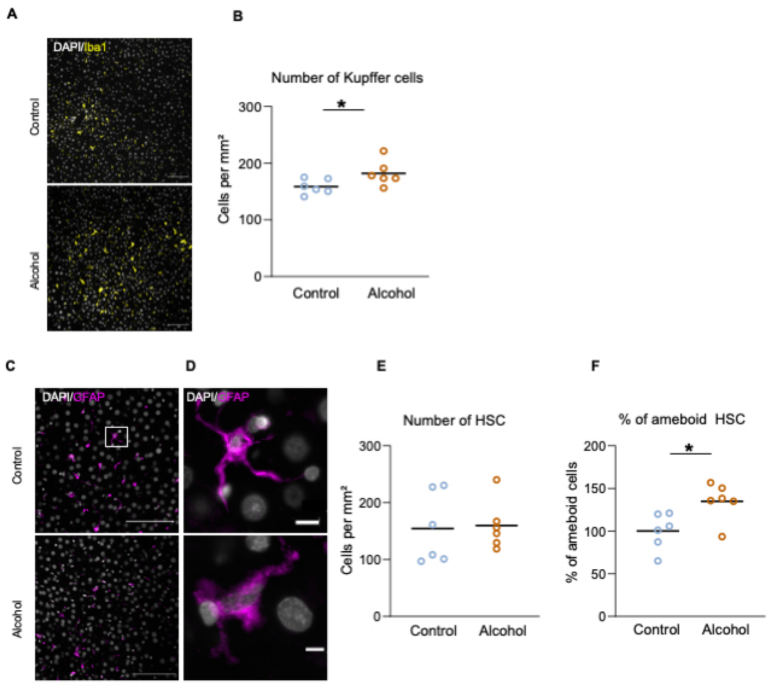

Figure 6- Chronic alcohol drinking provokes liver inflammation in sP rats.

210x290mm (87 x 87 DPI)

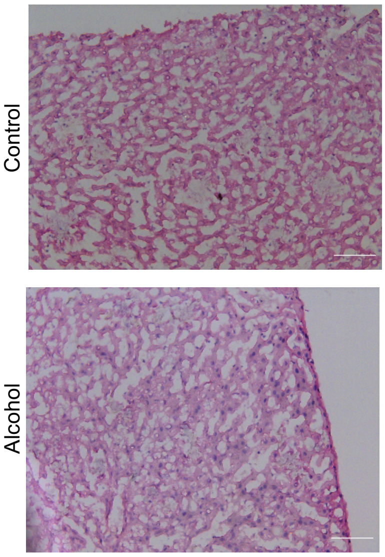

Supplementary figure 1: Hematoxylin & Eosin staining in liver of control and alcohol-drinking sP rats.

Representative photomicrographs of liver after hematoxylin & eosin staining. Scale bar 100µm.

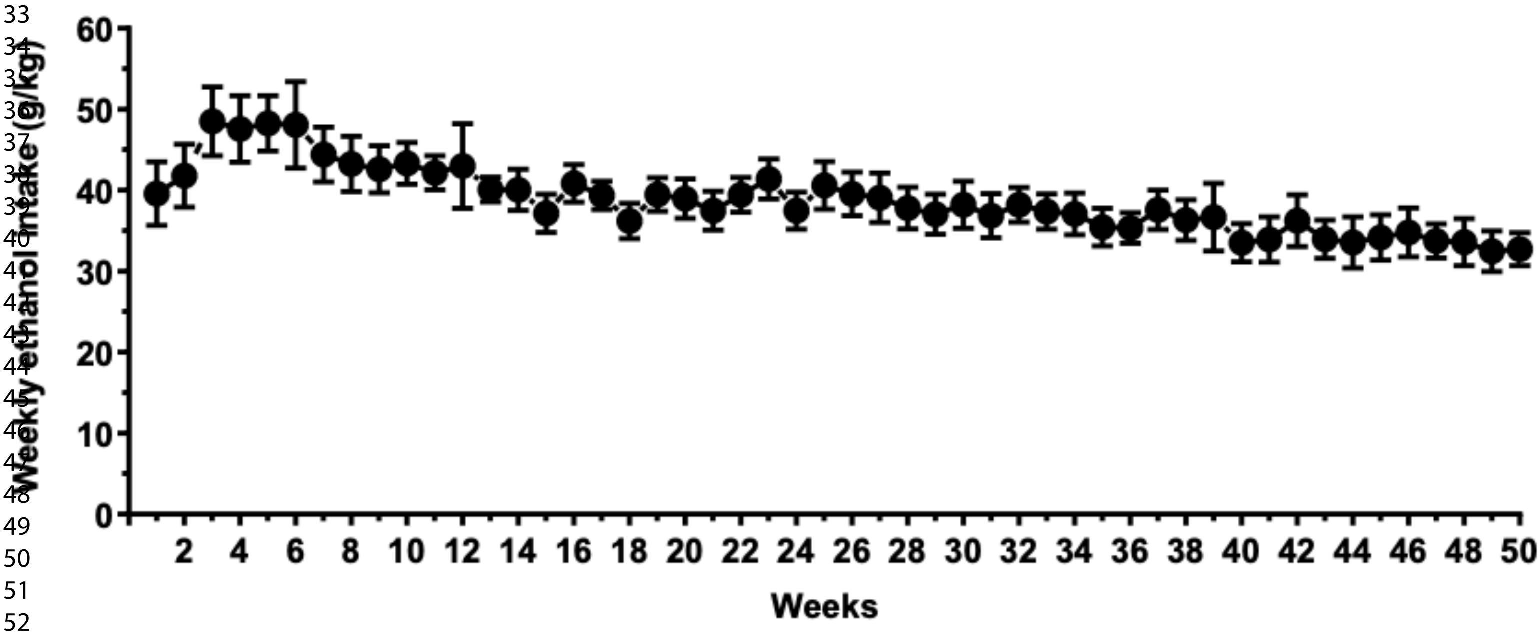

Supplementary figure 2: Weekly alcohol drinking pattern in sP rats.

Weekly alcohol intake in sP rats exposed to the standard, homecage 2-bottle "alcohol (10% v/v) *vs* water" choice regimen with unlimited access (24 hours/day) for 50 consecutive weeks. Weekly alcohol intake is expressed in g/kg pure alcohol. Each point is the mean ± SEM of n=6 rats. $F(49,245)=6.53$, $P<0.005$ (1-way ANOVA with repeated measures).
