# Supplementary material for: Role of inflammation in alcohol-related brain abnormalities: a translational study
Source: Brain Commun. 2021 Jul 16;3(3):fcab154. doi: 10.1093/braincomms/fcab154 (PMC8361421; doi:10.1093/braincomms/fcab154)
Supplement: fcab154_Supplementary_Data [file fcab154_supplementary_data.zip › Supplementary_figures.pdf]

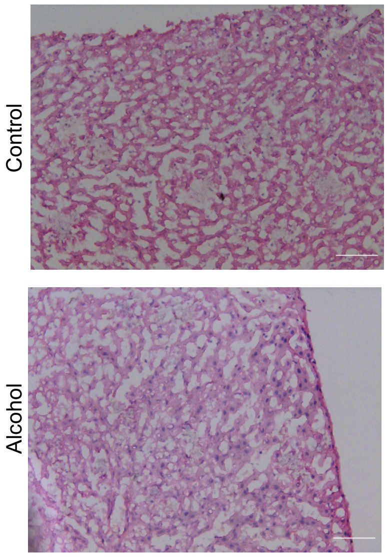

Supplementary figure 1: Hematoxylin & Eosin staining in liver of control and alcohol-drinking sP rats.
Representative photomicrographs of liver after hematoxylin & eosin staining. Scale bar 100µm.

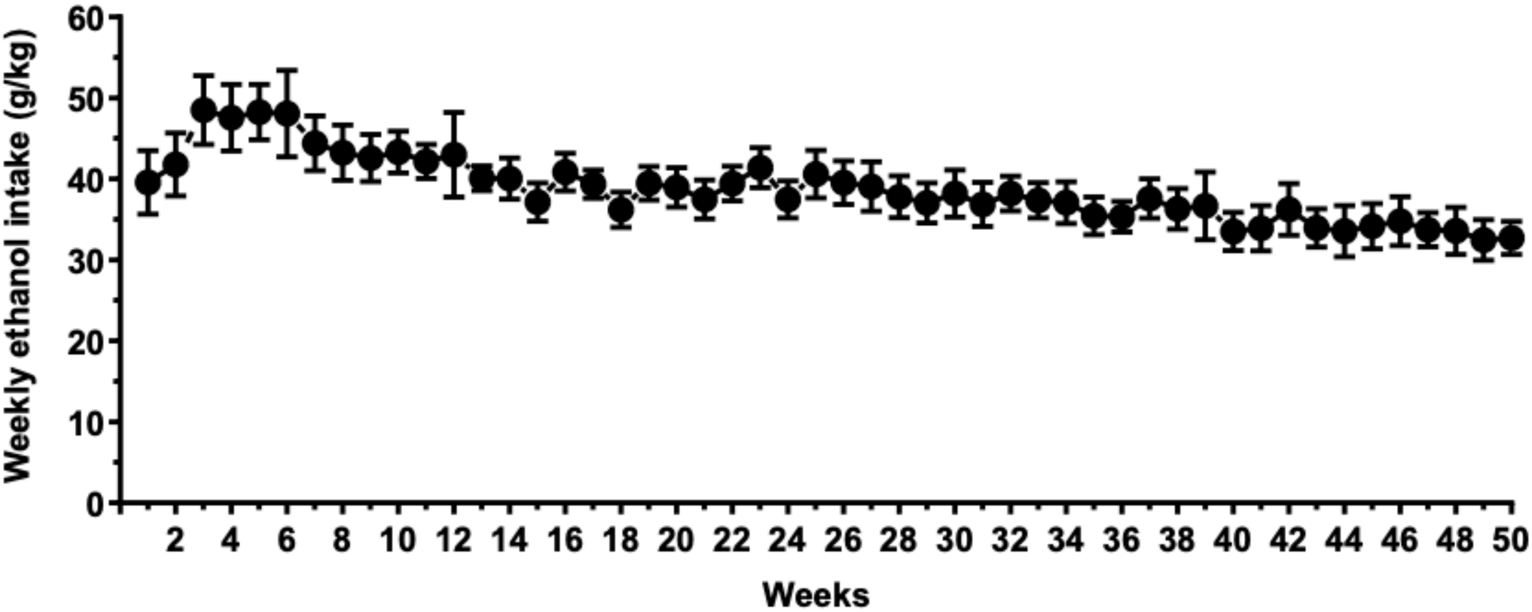

Supplementary figure 2: Weekly alcohol drinking pattern in sP rats.
Weekly alcohol intake in sP rats exposed to the standard, homecage 2-bottle "alcohol (10% v/v) *vs* water" choice regimen with unlimited access (24 hours/day) for 50 consecutive weeks. Weekly alcohol intake is expressed in g/kg pure alcohol. Each point is the mean ± SEM of n=6 rats. $F(49,245)=6.53$, $P<0.005$ (1-way ANOVA with repeated measures).
