# Supplementary material for: Role of inflammation in alcohol-related brain abnormalities: a translational study
Source: Brain Commun. 2021 Jul 16;3(3):fcab154. doi: 10.1093/braincomms/fcab154 (PMC8361421; doi:10.1093/braincomms/fcab154)
Supplement: fcab154_Supplementary_Data [file Original submission - authors response.pdf › Original submission - authors response.pdf]

We thank the editor and the two reviewers for their positive comment and careful review, which will help to improve the manuscript. Please find our answers to comments in black as well as suggested text changes in highlighted yellow.

Editorial office comments:

- Please add methodological details for the rat IHC experiments. How many sections were used? How were they selected? were they from the same level in all rats and taken at regular distances through the structure of interest (cc, liver, etc)?

We added details at the end of the methodological section (page 13) for the rat immunohistochemistry experiment: "Liver sections were taken from the left lateral lobe in all animals. Coronal brain sections (10µm-thick) containing the corpus callosum, cortex and/or hippocampus (coordinates: bregma -2 to -4.8 mm according to Paxinos-Watson (Paxinos and Watson, 2013)) were collected on gelatinized slices. For all the structures of interest, at least 5 images in 1 or 2 different sections per animal were analysed"

-For each statistical test, please report in the figure legend, the N, test used, relevant test statistic, and exact p values (for example F or t values for ANOVAs and t-tests)

Done.

-Please supply a graphical abstract. A graphical abstract is a visual representation of the central finding or methodology of the paper. It will be displayed in the table of contents. Authors should strive to make the graphical abstract informative, interesting, visually appealing, and straightforward (no caption needed, no abbreviations except gene names). Please upload as a separate high resolution image file (tiff preferred). The technical requirements for the graphical abstracts are: --size of the submitted image: 1200 pixels square at 300dpi --font of the text: Arial 12-16 point (smaller fonts will not be legible online)

A graphical abstract has been uploaded.

-Please add an Abbreviated Summary (up to 50 words) of your paper that captures the main purpose and conclusions of the work. The summary should state the main results written in the third person ('[1st author surname] et al. report that ...') and conclusion. This summary will be used in the contents list online and should not contain any abbreviations other than those on the accepted abbreviations list and accepted gene/protein names, making it accessible to the non-specialist. Please upload it as a Word document. Allowed abbreviations: AIDS; ANOVA;

AMPA; ATP; A,T,C,G; CNS; CSF; CT; DNA; ECG; EEG; EMG; GABA; HIV; MRI; NMDA; PET; PCR; RNA.

Abbreviated summary (page 2):

Lanquetin et al. report that systemic inflammation correlates with liver and brain abnormalities in alcohol use disorder patients. In alcohol-exposed rats, brain damage is associated with neuroinflammatory responses and liver inflammation. Chronic alcohol consumption results in peripheral inflammation, which alters the inflammatory status of the brain and liver in cascade.

-Please, add the scale bar to Fig. 3 a&b

Done.

-Please, add appropriate error bars to all Figures with data points

Done.

-Please, add units and descriptions on the Y-axis in Figure 1 (all data panels)

We added error bars (SEM) to all figures as well as units and descriptions on the Y-axis in Figure 1.

Reviewer n°1:

Control and AUD subjects: Subjects are nicely balanced across education, and other measures except large differences in daily alcohol consumption and AUDIT scores. The authors should add a measure of body size since brain volumes are larger in larger individuals.

We thank the reviewer for this suggestion. BMI data have been included in table 1.

In order to correct the potential effect of larger brain size on the brain volumes analysis, we have used the total intracranial volume as a covariate in brain volumes analysis, as it is usually done in neuroimaging studies.

Figure 1. AUD subjects show significant increases with considerable variability in TNF, MIP1, MCP1 and IL8 with many subject have values near control means. These are representative proinflammatory genes. It would add to the data to know if markers correlate with each other across subjects and/or with aspects of the AUD scores, daily consumption or time of abstinence.

This is now reported in the new version of the manuscript (page 15).

"AUD patients also had elevated levels of TNF and IL-8 compared to HC (p=0.02 and p=0.009 respectively; Fig. 1E). None of the cytokines correlated with each other in AUD patients. There was no significant correlation between elevated circulating cytokines (IL-8 and TNF) and alcohol-related variables either: AUDIT (p=0.79 and p=0.36), alcohol misuse (p=0.17 and p=0.08), daily alcohol consumption (p=0.38 and p=0.58), and Cushman score (withdrawal severity score; p=0.86 and p=0.53) in AUD patients."

Blood was collected during detoxification. Length of sobriety varied among AUD. A comment on withdrawal symptoms, duration of sobriety and time of cytokine measure would be interesting.

As mentioned in page 8 of the manuscript, "blood samples were collected for all participants, either at inclusion (healthy controls) or the day after admission to hospital (AUD patients) (24h) to ensure that patients were free from alcohol and to minimize the potential confusing effect of withdrawal." At this time, AUD patients did not present physical symptoms of withdrawal as assessed by the Cushman's scale (score<= 2; Cushman *et al.*, 1985).

Correlational analyses between the cytokines level on the one hand and alcohol-related variables on the other hand did not reveal any significant relationship (page 15).

A discussion of immune correlations and the different brain regions have negative correlations with the fibrosis score or IL8 with the white matter volume, whereas TNF shows positive correlations would add if there are thoughts.

We thank the reviewer for this suggestion. A comment on this has been added in the discussion section of the new version of the manuscript, pages 20-21:

"Positive and negative immune correlations have also been found in other neurological diseases such as schizophrenia (Fu et al 2019 ; Prasad et al 2015) or Alzheimer's disease (Swardfager et al 2017) indicating neuroinflammation with microglial activation. IL8 and TNF levels correlate with grey matter volume in regions typically impaired in AUD: prefrontal cortex (with orbitofrontal cortex), temporal cortex and cerebellum."

Post-mortem human AUD brain has been found to have increases in microglial markers. (He and Crews, Exp Neurol 2008; 210(:349-58) as well as increased expression of IL8 (Liu et al 2020. Hippocampal TNF-death receptors, caspase cell death cascades, and IL-8 in alcohol use disorder Molecular Psychiatry (2020)).

These articles are now cited in the discussion section and included in the reference list.

Introduction.

Pg. 6 lines 3-10: However, the study of neuroinflammation in AUD patients by positron emission tomography (PET) using TSPO ligand (used to evaluate microglial activation in vivo) has shown controversial results, with either lower or equal levels of neuroinflammation in AUD patients compared to controls (Kalk et al., 2017; Kim et al., 2018; Tyler et al., 2019). The authors could consider adding to this comment a recent study finding increased TSPO expression in post mortem AUD brain. Increased transcription of TSPO, HDAC2, and HDAC6 in the amygdala of males with alcohol use disorder. De Carvalho. Brain and Behavior ·December 2020

We thank the reviewer for this comment. The following sentence has been added in the introduction section of the new version of the manuscript (page 6):

"The study of neuroinflammation in AUD patients using positron emission tomography (PET) and TSPO ligand (used as an in vivo biomarker of microglial activation) has shown controversial results, with either lower or equal levels of neuroinflammation in AUD patients

compared to controls (Kalk et al., 2017; Kim et al., 2018; Tyler et al., 2019). Very recently, De Carvalho et al (2020) showed, for the first time, an increased transcription of TSPO, HDAC2 and HDAC6 in the amygdala of AUD patients suggesting a particular vulnerability of this region to neuroinflammation."

Reviewer n°2

Introduction:

- *hippocampus is sometimes considered distinct from the neocortex, but I would not consider it subcortical. Cingulate cortex is definitely not a subcortical structure.*

Following the reviewer's comment, we have modified the sentence in the new version of the manuscript as follows: "In cortical and subcortical regions, atrophy of the hippocampus (Wilson et al., 2017), mammillary bodies, cingulate cortex and thalami (Pitel et al., 2012; Segobin et al., 2019) have also been reported.*"*

- *The following sentence is confusing (p.5, first paragraph): "AUD patients have shown microstructural alterations ..., even when no structural MRI abnormalities were observed"*

This sentence is now: "AUD patients have shown microstructural alterations …, even when no macrostructural MRI abnormalities were observed.*"*

Methods:

- *The MRI data processing explanation is confusing:*

"The WM microstructural integrity mask was obtained by taking the FA maps of HC normalized to the MNI space, averaging them, and thresholding the resultant mean image at 0.3" Are the authors thresholding the FA? If so, this is not a clean way. FA can be <0.3 and still be white matter, for instances, in areas of crossing fibres. Having a high resolution T1 (and a low res T2, which is the DTI at b=0), I would recommend segmentation algorithms as implemented in SPM, FSL, etc.

We apologize for this mistake in reporting the methodology. The white matter mask for analyzing the FA values is the same as the one used for analyzing WM volumes. The text has been modified as follows:

"The white matter mask was obtained via the same procedure as for the GM mask, that is, by using unmodulated WM images from the HC group in MNI space, averaging them and thresholding the resultant mean image at 0.5. The same WM mask was used when analysing both, WM volumes and FA values respectively, while GM mask was used when analysing GM volumes."

"Voxel-based t-tests were conducted in SPM12 to compare HC and AUD on GM volume, WM volume and WM integrity (FA values)."

I might have overlooked something, but how can the authors with this methodology compare volume in a voxel-wise manner? What is being compared voxel by voxel? I understand voxel-wise comparisons in a Volume Based Morphometry analysis, but this is not what is being described here

The voxel-based t-tests were conducted separately for each measurement: (1) gray matter volume; (2) white matter volume; and (3) FA values. For gray and white matter volumes, we used maps that were modulated by the Jacobian determinants so that the voxel values represent tissue volumes (which are preserved using this method). Our statement probably lacked clarity. The voxel-based comparisons of the FA values represent alterations of white matter microstructural integrity, and not volume. This has been re-phrased in the new version of the manuscript accordingly (page 9).

In the rat study: "DTI data were acquired with the following parameters: TR 3500 ms, TE 23 ms, 32 diffusion directions,b=1048s/mm" B 1000 for ex vivo is quite low because diffusivity is reduced, so normally a larger b-value is needed to get a comparable contrast vs. in vivo. This is not critical here, and the results are of course valid, but this fact should be acknowledged somewhere in the manuscript. In vivo and ex vivo DTI results cannot be directly compared.

We acknowledge that the diffusivity contrast should not be directly compared and so we have added this comment in the "Chronic alcohol exposure induced similar brain abnormalities in a rat model of AUD " part of the discussion (page 22):

"It should be noted that the water diffusivity is reduced ex-vivo and that the diffusion images obtained in MRI cannot be compared directly between the AUD patients and the sP rats. To overcome this issue, we do not directly compare the raw diffusion values but the patterns of brain abnormalities in the different groups."

For both, rats and humans, TBSS (tract-based spatial statistics) analysis to compare FA values is the current standard procedure. I'd strongly recommend to perform this analysis.

We thank the reviewer for this comment, on which we completely agree. The reason why we did not use a voxel-based analysis method (VBM or TBSS) is that the RF receiver coils we had for imaging the ex-vivo samples were not what is tailored for subsequent voxel-based analyses of these animals. The volumetric receiver coil we had was too big for scanning the samples and the surface-coil used created "graded" signals, where it was tricky to correct for magnetic inhomogeneities. In summary, imaging and processing of these samples in the most optimal

way was quite an intensive labor. The brain extraction, segmentation and warping algorithms available did not provide a reliable result and we therefore dropped our initial idea of performing similar voxel-based analyses across our translational data. We therefore opted, for the rats' study, for measurements through manual delineation as this was the most robust method available to us. The dataset in humans has already been analyzed with TBSS before (Segobin et al., (HBM 2015)). Compared with TBSS, significant clusters from VBM studies are mathematically "closer" to ROI measurements, as they are both a reflection of the average value within that cluster. On the contrary, TBSS measurements come from a skeletonized dataset where data across a tract is effectively one-voxel thick and takes the maximum value across the perpendicular projection for each voxel (Smith et al., 2006). Hence, we preferred a voxel-based non-TBSS approach in our analyses

Results:

-Please report full statistics (i.e. F, t, values)

Done.

-Why a cluster size of 60 voxels is used to somehow correct the multiple-comparison uncorrected statistics?

The extent threshold of 60 is not what is used for correction for multiple comparisons. It represents the minimum size of a cluster (in terms of the number of voxels) that is subsequently required for statistical significance. There is no gold standard for the choice of extent threshold. However, it should be carefully chosen according to the size and morphology of brain regions, and is hence study specific. We have found that using k=60, which corresponds to 200 mm3 in volume (each voxel size being 1.5 x 1.5 x 1.5 mm^3), is large enough to avoid clusters that could have gained statistical significance through noise or artefactual results at the borders of brain regions, while preserving alterations to clusters within regions that are small or have a small cross-sectional area (e.g hippocampus, corpus callosum).

Statistical analyses are reported at $p<0.001$ uncorrected for multiple comparisons, which is quite usual for correlations.

-In the rat study (Figs 3-5), there is no indication of corrections for multiple comparisons. For instance, in Fig. 3, four different ROIs are being compared, in Fig. 4 there are 5 comparisons and in Fig. 5, 3 comparisons.

We did not perform any corrections for multiple comparisons in the rat study because of the lack of power due to the small sample size. We added this information in the legend of figure 4.

Discussion:

The limitations section should include also technical limitations.

We have added a paragraph in the discussion section (page 23) of the new version of the manuscript:

"Limitations, methodological considerations and perspectives

The similarities in the results concerning white matter abnormalities in the AUD patients and chronic alcohol-drinking rats are one of the major findings of this study. However, it should be noted that we have analyzed *in vivo samples* in Human and *ex vivo* samples in rats, and that there are significant basal differences in water diffusion and cellular death. Recent studies have shown that *ex vivo* use of diffusion MRI are comparable with human and preclinical models *in vivo* (Roebroeck et al. 2018; Walker et al. 2019). Ideally, tract-based spatial statistics (TBSS) would have been the method of choice to analyze both the animal and human datasets. However, for the animal dataset, the brain extraction, segmentation and warping algorithms available did not provide a reliable result, making a voxel-based analysis impossible. The best and most robust option available was thus through manual delineation. The dataset in humans has already been analyzed with TBSS before (Segobin et al., (HBM 2015)). Compared with TBSS, significant clusters from VBM studies are mathematically "closer" to ROI measurements, as they are both a reflection of the average value within that cluster. On the contrary, TBSS measurements come from a skeletonized dataset where data across a tract is effectively one-voxel thick and, numerically, takes the maximum value across the projection perpendicular to the local skeleton structure for each voxel within the skeleton (Smith et al., 2006). Hence, we preferred a voxel-based non-TBSS approach in our analyses for the human dataset."
