# Supplementary material for: Role of inflammation in alcohol-related brain abnormalities: a translational study
Source: Brain Commun. 2021 Jul 16;3(3):fcab154. doi: 10.1093/braincomms/fcab154 (PMC8361421; doi:10.1093/braincomms/fcab154)
Supplement: fcab154_Supplementary_Data [file Original submission - decision letter.pdf › Original submission - decision letter.pdf]

Original Submission-Decision Letter

09-Feb-2021

Dear Dr. Pitel,

Thank you for your recent submission BRAINCOM-2021-016 entitled "Role of inflammation in alcohol-related brain abnormalities: a translational study" to Brain Communications.

Your manuscript has been reviewed, and while the reviewers and editorial team appreciate many aspects of the paper, they raise some concerns that you will need to address in order for the paper to be considered for publication.

If you can address these concerns, I invite you to submit a revised manuscript and a response to each of the reviewers' comments. The reviewer(s)' comments are included at the bottom of this message.

In addition to the reviewers' comments, please note our editorial comments added below to comply with our requirements for presentation of scientific data at Brain Communications.

Please format your revised manuscript according to our Instructions to Authors https://academic.oup.com/braincomms/pages/General_Instructions – a summary of key points is attached to help with this.

To revise your manuscript, click the link below or log into https://mc.manuscriptcentral.com/braincom and enter your Author Centre, where you will find your manuscript title listed under "Manuscripts with Decisions." Under "Actions," click on "Create a Revision." Your manuscript number has been appended to denote a revision.

*** PLEASE NOTE: This is a two-step process. After clicking on the link, you will be directed to a webpage to confirm. ***

https://mc.manuscriptcentral.com/braincom?URL_MASK=da59c5b7e409491e94c33d551d9e048b

Please upload a revised manuscript with changes tracked with track changes or in coloured text.

When submitting your revised manuscript, you will be asked to respond to the reviewer(s) comments. Please be as specific as possible in your response to all of the comments.

The editorial team at Brain Communications aims to increase transparency in scientific publishing and to demonstrate the value that peer review brings to the scientific process. Please therefore consider whether you are willing to have reviewer(s)' comments on your paper and your response published as supplementary data with the paper if it is accepted. This is entirely optional and will only be done if both the authors and reviewers agree. While submitting your revision there will be an opportunity to review or update the answer you gave previously, if necessary.

IMPORTANT: Your original files are available to you when you upload your revised manuscript. Please delete any redundant files before completing the submission.

Because we are trying to facilitate timely publication of manuscripts submitted to Brain Communications, we would appreciate your revised manuscript as soon as possible. If it is not possible for you to resubmit within 3 months, the option to submit a revision will expire, and we would then consider your revised paper as a new submission. However, if you need a little more time, please get in touch and we can extend this deadline for you.

Once again, thank you for submitting your manuscript to Brain Communications and I look forward to receiving your revision.

Yours sincerely,

Dr. David Belin
Editor, Brain Communications

Reviewer(s)' Comments to Author:

Reviewer: 1 (Crews, Fulton)

Comments to the Author
BRAINCOM-2021-016
Role of inflammation in alcohol-related brain abnormalities: a translational study
Journal: Brain Communications

The studies reported here are interesting and contribute to understanding of AUD pathology. In recently abstinent and non-cirrhotic AUD patients liver fibrosis is found to positively correlate with subcortical regions volumes (especially in right and left putamen) and level of circulating proinflammatory cytokines. In a model of AUD, sP rats, alcohol caused microstructural white matter and grey matter changes to those found in AUD patients. Results in both humans and rats support a role for peripheral innate immune cytokines with pathology in brain and liver caused by chronic alcohol consumption. These findings are interesting and may lead to new new therapeutics. However, the manuscript could be improved by addressing the following:

Control and AUD subjects: Subjects are nicely balanced across education, and other measures except large differences in daily alcohol consumption and AUDIT scores. The authors should add a measure of body size since brain volumes are larger in larger individuals.

Figure 1. AUD subjects show significant increases with considerable variability in TNF, MIP1, MCP1 and IL8 with many subject have values near control means. These are representative proinflammatory genes. It would add to the data to know if markers correlate with each other across subjects and/or with aspects of the AUD scores, daily consumption or time of abstinence. Blood was collected during detoxification. Length of sobriety varied among AUD. A comment on withdrawal symptoms, duration of sobriety and time of cytokine measure would be interesting.

A discussion of immune correlations and the different brain regions have negative correlations with the fibrosis score or IL8 with the white matter volume, whereas TNF shows postitive correlations would add if there are thoughts.

Post-mortem human AUD brain has been found to have increases in microglial markers. (He and Crews, Exp Neurol 2008; 210(:349-58). As well as increased expression of IL8 (Liu et.al. . Hippocampal TNF-death receptors, caspase cell death cascades, and IL-8 in alcohol use disorder Molecular Psychiatry (2020).

Introduction.
Pg. 6 lines 3-10: However, the study of neuroinflammation in AUD patients by positron emission tomography (PET) using TSPO ligand (used to evaluate microglial activation in vivo) has shown controversial results, with either lower or equal levels of neuroinflammation in AUD patients compared to controls (Kalk et al., 2017; Kim et al., 2018; Tyler et al., 2019).
The authors could consider adding to this comment a recent study finding increased TSPO expression in post mortem AUD brain. Increased transcription of TSPO, HDAC2, and HDAC6 in the amygdala of males with alcohol use disorder. De Carvalho. Brain and Behavior ·December 2020

Reviewer: 2

Comments to the Author
In this study, the authors look for correlations between brain structural alterations and the inflammatory profile in AUD patients and an animal model of AUD (sP rats). The study is focused on the liver-brain axis in the context of AUD. The topic is very important, and the work is timely. The results are interesting and promising, but there are some limitations that decreased the strength of the work (i.e. technical limitations and statistical methods).

Introduction:
- hippocampus is sometimes considered distinct from the neocortex, but I would not consider it subcortical. Cingulate cortex is definitely not a subcortical structure.
- The following sentence is confusing (p.5, first paragraph): “AUD patients have shown microstructural alterations …, even when no structural MRI abnormalities were observed”

Methods:
- The MRI data processing explanation is confusing:

"The WM microstructural integrity mask was obtained by taking the FA maps of HC normalized to the MNI space, averaging them, and thresholding the resultant mean image at 0.3”
Are the authors thresholding the FA? If so, this is not a clean way. FA can be <0.3 and still be white matter, for instances, in areas of crossing fibres. Having a high resolution T1 (and a low res T2, which is the DTI at b=0), I would recommend segmentation algorithms as implemented in SPM, FSL, etc.

"Voxel-based t-tests were conducted in SPM12 to compare HC and AUD on GM volume, WM volume and WM integrity (FA values).”
I might have overlooked something, but how can the authors with this methodology compare volume in a voxel-wise manner? What is being compared voxel by voxel?
I understand voxel-wise comparisons in a Volume Based Morphometry analysis, but this is not what is being described here.

In the rat study: "DTI data were acquired with the following parameters: TR 3500 ms, TE 23 ms, 32 diffusion directions,b=1048s/mm"
B 1000 for ex vivo is quite low because diffusivity is reduced, so normally a larger b-value is needed to get a comparable contrast vs. in vivo. This is not critical here, and the results are of course valid, but this fact should be acknowledged somewhere in the manuscript. In vivo and ex vivo DTI results cannot be directly compared.

- For both, rats and humans, TBSS (tract-based spatial statistics) analysis to compare FA values is the current standard procedure. I'd strongly recommend to perform this analysis.

Results:
- Please report full statistics (i.e. F, t, values)
- Why a cluster size of 60 voxels is used to somehow correct the multiple-comparison uncorrected statistics?
- In the rat study (Figs 3-5), there is no indication of corrections for multiple comparisons. For instance, in Fig. 3, four different ROIs are being compared, in Fig. 4 there are 5 comparisons and in Fig. 5, 3 comparisons.

Discussion:
The limitations section should include also technical limitations.

Editorial office comments:
- Please add methodological details for the rat IHC experiments. How many sections were used? How were they selected? were they from the same level in all rats and taken at regular distances through the structure of interest (cc, liver, etc)?

-For each statistical test, please report in the figure legend, the N, test used, relevant test statistic, and exact p values (for example F or t values for ANOVAs and t-tests)

-Please supply a graphical abstract. A graphical abstract is a visual representation of the central finding or methodology of the paper. It will be displayed in the table of contents. Authors should strive to make the graphical abstract informative, interesting, visually appealing, and straightforward (no caption needed, no abbreviations except gene names). Please upload as a separate high resolution image file (tiff preferred). The technical requirements for the graphical abstracts are:
--size of the submitted image: 1200 pixels square at 300dpi
--font of the text: Arial 12-16 point (smaller fonts will not be legible online)

-Please add an Abbreviated Summary (up to 50 words) of your paper that captures the main purpose and conclusions of the work. The summary should state the main results written in the third person ('[1st author surname] et al. report that …') and conclusion. This summary will be used in the contents list online and should not contain any abbreviations other than those on the accepted abbreviations list and accepted gene/protein names, making it accessible to the non-specialist. Please upload it as a Word document. Allowed abbreviations: AIDS; ANOVA; AMPA; ATP; A,T,C,G; CNS; CSF; CT; DNA; ECG; EEG; EMG; GABA; HIV; MRI; NMDA; PET; PCR; RNA.

-Please, add the scale bar to Fig. 3 a&b

-Please, add appropriate error bars to all Figures with data points

-Please, add units and descriptions on the Y-axis in Figure 1 (all data panels)
